# Diagnostic and metabolic insights into secondary lactose intolerance in infants via fecal lactose quantification and gut microbiome profiling

**DOI:** 10.3389/fimmu.2026.1711945

**Published:** 2026-04-21

**Authors:** Jian Kuang, Mengmei Zhang, Xiangyu Bian, Xin Wang, Xiaoqiong Li, Qingbin Wu, Jinjun Li

**Affiliations:** 1 State Key Laboratory for Managing Biotic and Chemical Threats to the Quality and Safety of Agro-Products, Institute of Food Science, Zhejiang Academy of Agricultural Sciences, Hangzhou, China; 2 Key Laboratory of Postharvest Preservation and Processing of Vegetables (Co-construction by Ministry and Province), Ministry of Agriculture and Rural Affairs, Hangzhou, China; 3 Zhejiang Key Laboratory of Intelligent Food Logistic and Processing, Zhejiang Academy of Agricultural Sciences, Hangzhou, China; 4 Department of Gastroenterology, Children’s Hospital of Soochow University, Suzhou, China

**Keywords:** secondary lactose intolerance, gut microbiota, lactose glycolysis metabolism, SCFAs, 16s rRNA, *in vitro* fermentation

## Abstract

**Background:**

Secondary Lactose intolerance (SLI) is common among infants in China, primarily resulting from secondary lactase deficiency due to mucosal damage. Current diagnostic methods are limited by poor sensitivity and specificity.

**Objective:**

To investigate gut microbial composition and metabolic dysfunction in infants with SLI and to explore the potential utility of residual fecal lactose as a non-invasive indicator related to SLI.

**Results:**

SLI infants exhibited significantly higher residual fecal lactose and lactate levels accompanied by reduced fecal short-chain fatty acid (SCFA) availability, consistent with incomplete lactose digestion and altered microbial fermentation. Microbiota profiling revealed marked depletion of *Bacteroidetes* and certain *Firmicutes* (e.g. *Ruminococcaceae, Erysipelotrichaceae, Peptostreptococcaceae, Megasphaera*), along with reduced glycolysis pathways. *In vitro* fermentation assays demonstrated a consistent reduction in total acid, acetate, and propionate production across multiple media, while lactate and gas production were significantly elevated in SLI samples under lactose, FOS, GOS, and starch-enriched conditions. Butyrate synthesis was partially preserved under protein-rich or minimal carbon media, indicating selective resilience of butyrogenic pathways. Microbial β-diversity analysis confirmed structural dysbiosis, with increased abundance of gas-associated taxa, including Clostridium.

**Conclusion:**

Residual fecal lactose, when interpreted alongside microbial and metabolic profiles, may serve as a non-invasive indicator associated with secondary lactose intolerance in infants. These findings delineate microbiota–metabolism features consistent with SLI pathophysiology and provide a conceptual framework for future validation studies and the development of nutritional or probiotic interventions.

## 1 Introduction

Lactose, the primary carbohydrate found in mammalian milk and dairy products, is a disaccharide consisting of glucose and galactose linked by a β-1,4-glucosidic bond. Human milk contains the highest lactose content, averaging around 7% (1), while whole milk typically contains about 4.8% lactose. In an infant’s diet, lactose contributes to 35-55% of the total calories consumed and serves as the primary energy source in early life (2). The digestion and absorption of lactose rely upon the presence of the proximal intestinal brush border enzyme known as lactase phlorizin hydrolase (LPH) (3, 4). In most mammals, the activity of intestinal LPH is high at birth and gradually declines after weaning, leading to a loss of lactose digestion capability over time (1). Insufficient secretion or reduced activity of LPH in the intestine leads to incomplete digestion and absorption of ingested lactose. This leads to an increase of lactose concentration within the intestinal cavity, higher permeability, resulting in osmotic retention of water in the intestine, which in turn leads to osmotic diarrhea (5). Furthermore, undigested lactose traverses into the colon, where it undergoes bacterial metabolism, generating lactic acid, SCFA, methane (CH_4_), hydrogen (H_2_), carbon dioxide (CO_2_), and other byproducts. Some of these byproducts are reabsorbed into the bloodstream, while the remainder accumulates in the intestinal cavity, further provoking intestinal peristalsis. This exacerbates diarrhea, accompanied by symptoms such as abdominal pain, distension, and discomfort (6, 7), collectively known as symptoms of lactose intolerance (LI). The global prevalence of LI averages around 65% of the total population, but this figure varies significantly across regions and ethnic groups. LI is most commonly observed in African Americans, Hispanics, Latinos, and Asians, while it is relatively rare among individuals of European descent (8). Interestingly, the incidence of LI among Chinese infants is notably high, ranging from 46.9% to 70%.

Currently, multiple diagnostic methods are available to identify lactose intolerance for adults and infants, which is shown as follows. For instance, genetic test enables the confirmation or exclusion of a primary LI and is characterized by its minimally invasive nature. However, it cannot detect any secondary LI from primary one (9). Hydrogen breath test (HBT) is the predominant method for diagnosing lactose intolerance, however, it is associated with disadvantages such as a long duration (3–6 hours) and the potential for false positives and false negatives (10). Infants often present challenges during HBT due to factors such as crying, hyperventilation, and other issues, making it unsuitable for use in small infants. The lactose tolerance test (LTT) is seldom conducted due to its low sensitivity and specificity. Variations in gastric emptying time among patients and subjective factors related to glucose metabolism contribute to the inconsistent and unreliable results obtained from this test (10, 11). Small intestinal biopsy for lactose or quick lactase test is an invasive procedure that poses challenges when performed on infants and young children (10, 12). Due to these difficulties and the reluctance of parents to consent to such procedures, its clinical application is infrequent. Furthermore, the degree of lactose breakdown can be assessed by analyzing stool reducing sugar levels and pH values (13). However, this method requires fresh stool specimens, which can be challenging to obtain from infants and young children (5). Additionally, interpreting reducing sugar readings is subjective and can be influenced by the color of the specimen, adding a further limitation to this approach. Moreover, Serum galactose or urine galactose test can serve as alternative methods for detecting lactose intolerance. It is important to note that the literature reports variable test performance in these methods, and false-positive results may occur in cases of rapid transit or other related conditions (14). In summary, there still lacks practical and effective diagnostic methods to help people determine whether a patient, especially for infants, is lactose intolerant or not. Given these limitations, there remains a pressing need for reliable, non-invasive, and infant-friendly diagnostic tools for identifying lactose intolerance, particularly the secondary form which is often triggered by intestinal infections, inflammation, or immature gastrointestinal development. Advances in microbiome research have revealed that undigested lactose entering the colon is subject to microbial fermentation, and that the composition and metabolic activity of gut microbiota play a crucial role in determining symptom severity (15, 16).

It is now recognized that key microbial metabolites, such as SCFAs, lactate, and fermentation gases, can reflect functional changes associated with carbohydrate malabsorption (17). Moreover, high-throughput sequencing of 16S rRNA genes enables precise characterization of gut microbial shifts, offering a promising avenue for mechanistic insight and potential biomarker development (18). Despite these advances, there is limited understanding of how microbial structure and metabolism are altered in infants with secondary lactose intolerance, and whether these features could be harnessed to inform diagnosis and intervention.

In this study, we adopt an integrative approach to characterize secondary lactose intolerance in infants by integrating fecal residual lactose quantification, *in vitro* fermentation assays, and gut microbiota profiling. Specifically, we aim to: (i) assess residual fecal lactose as a non-invasive biomarker for SLI; (ii) investigate alterations in gut microbial composition and glycolytic capacity in SLI infants compared with healthy controls; and (iii) explore microbial metabolic responses to various fermentable substrates, including lactose, oligosaccharides, and proteins. Through this integrative approach, we aim to provide a conceptual and mechanistic framework for understanding microbial and metabolic alterations associated with SLI and to inform future validation studies and nutrition- or microbiota-oriented intervention strategies.

## 2 Materials and methods

### 2.1 Patients and study design

**Figure 1:**
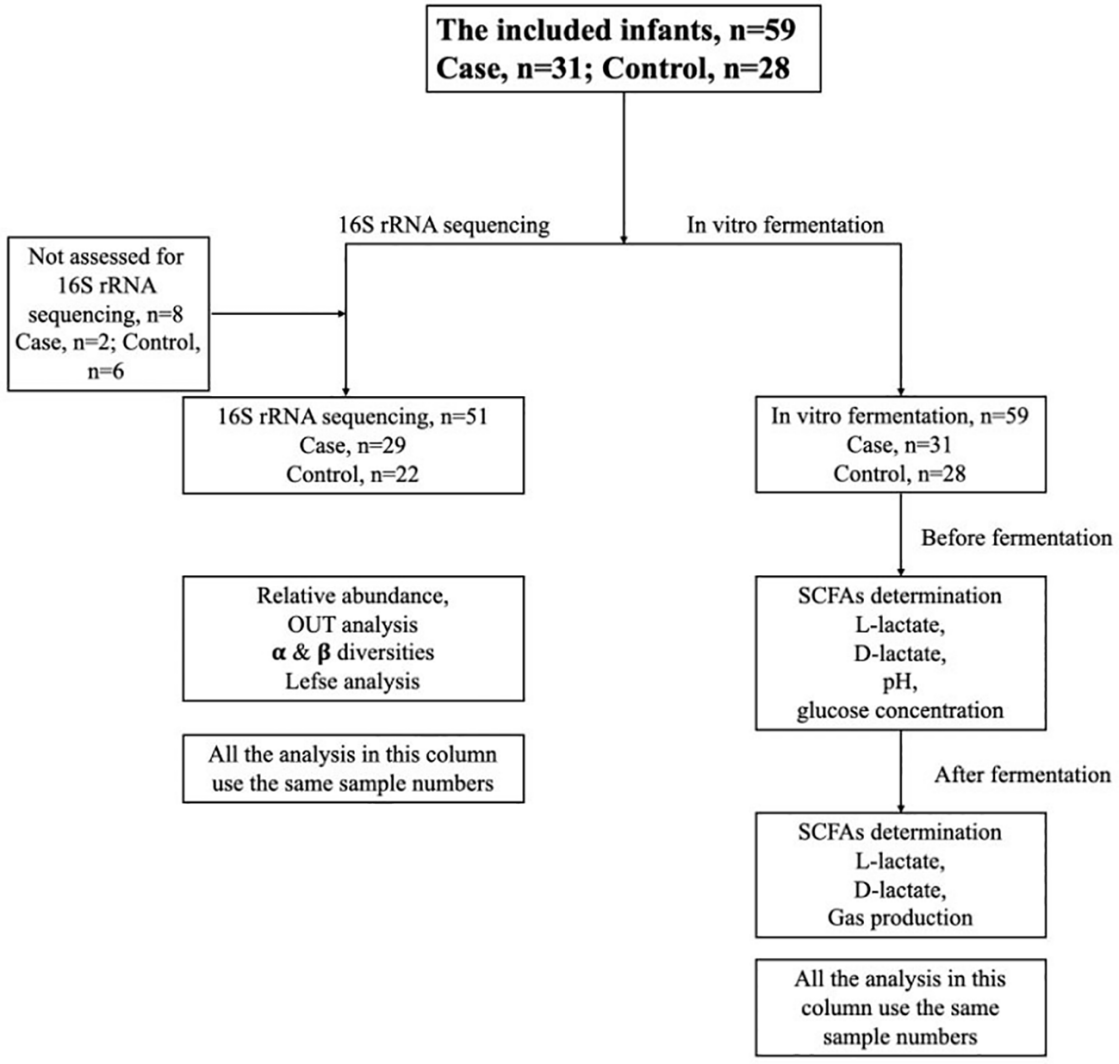
Participant flow chart.

A total of 59 subjects were selected into two groups: Cases (n=31) and controls (n=28), which was shown in **Figure 1**. Infants with secondary lactose intolerance with diarrhea, colic and abdominal distension symptoms in cases were recruited from the outpatient and inpatient departments of the Department of Gastroenterology of the Children’s Hospital of Soochow University from March 2021 to November 2021 with an average of 4.77 (3.20, 8.76) months, 11 males and 20 females. Among them, 27 cases were mainly breastfed or breastfed, and 4 cases were fed formula milk or mainly formula milk. The following criteria must be met at the same time (1): Infants were within 1 year of age, and the growth and development were basically normal that refers to meeting the WHO child growth standards (19) (2); Infants were clinical symptoms such as diarrhea, abdominal distension, and intestinal colic (3); The gestational age was 37~42 weeks, and the delivered weight was within the range of 2500~4000 g (4); There was no delay in meconium discharge, and the frequency and characteristics of previous stool were normal. Meanwhile, 28 healthy infants under 1 year old who underwent routine physical examination in a community in Suzhou were selected as the control group, with an average age of 5.50 (3.77, 8.00) months, 14 males and 14 females. Among them, 20 cases were mainly breastfed or breastfed, and 8 cases were fed formula milk or mainly formula milk and the enrollment criteria were as follows (1): Within 1 year of age, and the growth and development were basically normal (2); The gestational age was 37 to 42 weeks, and the delivered weight was within the range of 2500~4000 g (3); There was no history of infectious diseases within 4 weeks. The exclusion criteria for research subjects were as follows (1): Gestational age less than 37 weeks or more than 42 weeks (2); Delivery weight less than 2500 g or more than 4000 g (3); Clearly diagnosed with bacterial intestinal infection or respiratory tract infection (4); Recent There was a history of antibiotic use within 4 weeks (5); There was a history of using lactase, lactose-free milk powder, and amino acid milk powder (6); There was obvious growth retardation (the length and weight were less than 2 standard deviations of infants of the same age) (7); There was gastric history of intestinal organic diseases and major congenital diseases (8); Family members refused to provide baby stool samples. Written informed consent was provided by the guardians of all the participants after they were informed of the method, purpose and operation procedures of this study in advance. The study was approved by the Ethics committee of the Children’s Hospital Soochow University (protocol number: 2021CS114). It was conducted according to the ethical standards specified by the above committees.

### 2.2 Collection of infant fecal samples

Inclusion criteria of stool samples were as follows (1): To avoid mixing urine during collection, the seven-step handwashing method (20) was taken and sterile gloves were worn before collecting fecal specimens (2); The amount of feces was greater than 15g and was collected by a sterile and dry fecal tube (3); Basic information was marked on the fecal tube including name, gender, number, collection time, and etc. (4); Stool samples were stored at 4°C and performed the following experiments within 2 hours.

### 2.3 Allocation of medium

The formulation of the YCFA basic medium formula (21) is as follows (g/L): Pancreas Peptone 10, yeast extract 2.5, L-cysteine hydrochloride 1, NaCl 0.9, CaCl_2_·6H_2_O 0.009, KH_2_PO_4_ 0.45, K_2_HPO_4_ 0.45, MgSO_4_·7H_2_O 0.09, Azurin (1 mg/mL) 1mL, Heme (5 mg/mL) 2 mL, vitamin I solution 200 μL. The vitamin I solution contains the following components (mg/mL): Biotin (VH) 0.05, cobalamin (VB12) 0.05, p-aminobenzoic acid 0.15, folic acid 0.25, pyridoxine (VB6) 0.75. All components were uniformly provided by Sigma Corporation, USA. To investigate the metabolism of gut microbiota, a total of 8 distinct medium was prepared. Each medium was supplemented with one of the following substrates: inulin (INU), lactose (LAT), lactulose (LAU), fructo-oligosaccharides (FOS), galacto-oligosaccharides (GOS), starch (STA), hydrolyzed casein (YCP), hydrolyzed whey protein (YLP). These substrates were chosen to evaluate their effects on the metabolic activity of gut microbiota, facilitating a comprehensive understanding of the interactions between different carbohydrates and protein sources in the gut ecosystem. After the culture medium was configured, the pH was adjusted to 6.5, and then 5 mL culture medium was divided into 10 mL bottles under anaerobic conditions, and then sealed and autoclaved.

### 2.4 pH analysis of fecal samples

The pH of the samples was calculated by a pH meter (PHB-4, Shanghai INESA Scientific Instrument Co., Ltd., China). In details, 5 g of fresh feces were exactly weighed with an electronic balance and transferred into the sample bottle. Subsequently, 20 mL of neutral distilled water was drawn into the bottle with a disposable syringe, and the sample was well shanked.

### 2.5 Diagnose lactose intolerance

To provide useful evidence for the methodology for diagnosing lactose intolerance, our group developed a method for indirect determination of residual lactose content in feces. In details, 10% of fecal suspension was prepared through weighing 0.3 g of fresh feces into a sample bottle and mixing well with 3 mL of purified water with a vortex mixer (Shanghai Xinyun Technology Co., Ltd., China). Subsequently, two centrifuge tubes (1.5 mL) named A and B were prepared, in which, 450 μL of the suspension and 50μL of purified water were accurately taken and mixed will in A tube which is called Glucose content in raw fecal (GCRF), and incubated at 37°C for 20 minutes; 450 μL of the suspension and 50 μL of lactase solution were accurately taken and mixed will in B tube which is called glucose content in raw fecal after enzymatic hydrolysis (GCRH), and incubated at 37°C for 20 minutes. Then, the glucose content of A and B tubes was detected and recorded by FB-21 Automatic Biochemical Analyzer (Shenzhen Hailu Biotechnology Co., Ltd., C) using the standard procedures described by the manufacturer. The calculated difference between the two tubes of glucose (NGC) can be used to represent the residual lactose content in stool.

### 2.6 16S rRNA gene amplicon sequencing and microbiota analysis

#### 2.6.1 Sample collection and DNA extraction

Methods are similar to the previous method with little modifications (22). Fecal samples were freshly collected from all infants using sterile tubes, immediately snap-frozen in liquid nitrogen, and stored at −80°C until DNA extraction. Microbial genomic DNA was extracted from approximately 200 mg of feces using the QIAamp Fast DNA Stool Mini Kit (Qiagen, Germany) following the manufacturer’s protocol. To enhance bacterial cell lysis, an additional mechanical disruption step was included using a bead-beating procedure with 0.1 mm zirconia/silica beads (MP Biomedicals, USA). The concentration and purity of extracted DNA were assessed using a NanoDrop 2000 spectrophotometer (Thermo Fisher Scientific, USA) and agarose gel electrophoresis.

#### 2.6.2 PCR amplification and library construction

The nearly full-length bacterial 16S rRNA gene (covering V1–V9) was amplified using the universal primers 27F (5′-AGRGTTYGATYMTGGCTCAG-3′) and 1492R (5′-RGYTACCTTGTTACGACTT-3′) were used. PCR reactions were performed in 25 µL volumes containing 12.5 µL of 2× KAPA HiFi HotStart ReadyMix (Roche), 0.2 µM of each primer, and ~10 ng of DNA template. The amplification protocol was as follows: initial denaturation at 95°C for 5 min; 25 cycles of 98°C for 20 s, 55°C for 15 s, and 72°C for 90 s; with a final extension at 72°C for 10 min. PCR products were purified using AMPure XP beads (Beckman Coulter, USA), and libraries were constructed using the Illumina TruSeq DNA PCR-Free Kit (Illumina, USA) according to the manufacturer’s instructions. Quality and quantity of libraries were assessed with the Agilent 2100 Bioanalyzer (Agilent Technologies, USA) and Qubit 3.0 fluorometer (Thermo Fisher Scientific, USA).

#### 2.6.3 Bioinformatics and statistical analysis

Sequencing was performed on the Illumina NovaSeq 6000 platform using a paired-end 250 bp (PE250) strategy, generating high-quality reads covering the near full-length 16S rRNA gene. Raw reads were quality filtered and merged using FLASH (v1.2.11), and chimeric sequences were removed using USEARCH (v11). Denoising, ASV inference, and feature table construction were conducted using the DADA2 pipeline implemented in QIIME2 (v2021.4). Taxonomic classification was performed using a Naive Bayes classifier trained on the SILVA 138 database (99% OTUs, full-length 16S rRNA gene sequences). Alpha diversity (Shannon, Chao1, and Simpson) and beta diversity (Bray–Curtis, Jaccard, weighted and unweighted UniFrac) metrics were calculated using QIIME2 (v2021.11). Community composition differences between groups were visualized by PCoA and assessed using PERMANOVA. Differentially abundant taxa were identified using LEfSe (LDA score > 2.0, p < 0.05). Microbial functional potential was predicted using PICRUSt2, with pathway enrichment analysis performed based on the MetaCyc database.

### 2.7 Fermentation of fresh infant fecal samples

A fecal specimen weighing 0.8 g was transferred into a sample tube and combined with 8 mL of phosphate-buffered saline (PBS) using a fecal processing instrument. The mixture was then thoroughly shaken to create a 10% fecal suspension. Subsequently, 0.8 mL of this suspension was aliquoted into a 1 mL centrifuge tube. From this 1 mL centrifuge tube, 500 μL of the fecal suspension was transferred to two separate new tubes. To one of these tubes, 100 μL of Barbital-Phosphate buffer was added, while the other tube remained without any additional substances. Each tube was labeled with a unique identification number, thoroughly shaken to ensure proper mixing, and subsequently stored at -20°C for further analysis. 0.50mL of fecal suspension was aspirated from the sample tube and inoculated into the following media: YCFA, LAT, LAU, FOS, GOS, INU, STA, YCP, YLP. The cultures were thoroughly mixed, and the initial air pressure (0h) was measured by a barometric pressure gauge. After 24 hours, the air pressure in each medium was measured again with a barometric pressure gauge, and the pressure difference between each media before and after was calculated and recorded. Subsequently, 0.8 mL of the fermentation broth from each medium was transferred into a 1mL centrifuge tube. A 500μL aliquot of the supernatant was pipetted into another centrifuge tube, followed by the addition of 100μL of a crotonic acid-metaphosphate solution. Each tube was labeled, mixed thoroughly, and stored at -20°C until future analysis. Additionally, 0.50 mL of fermentation broth from the YCFA and LAT media after 24 hours was collected into 1.5 mL centrifuge tubes and stored at −20°C for future test.

### 2.8 Determination of short-chain fatty acids

The content of SCFAs, including Acetic acid, Propionic acid, Butyric acid, Isobutyric acid, Valeric acid, and Isovaleric acid, in the samples before and after fermentation were determined by gas chromatography-mass spectrometry (Agilent Technologies, Santa Clara, CA, USA) using the same method as described in the previous experiment (21, 23) with minor modifications. Briefly, the samples were subjected to centrifugation at 4°C, 11000 r/min for 3 min, followed by filtration of the supernatant using a 0.22 µm filter. After filtration, 150 μL of the supernatant was transferred into a sample vial for analysis, ensuring the removal of any bubbles at the bottom of the inner tube. The GC-MS system utilized a GC9720II gas chromate graph (Fuli, Inc., Zhejiang, China) equipped with a flame ionization detector and an Agilent-FFAP column (temperature range: 75–220°C, dimensions: 30 m ×0.25 mm ×0.25 μm). Nitrogen (N_2_) served as the carrier gas, with a constant flow rate set at 20 mL/min and a splitting ratio of 1:10. The flow rates of hydrogen (H_2_) and air through the column were maintained at 40 mL/min and 400 mL/min, respectively. The column temperature was initially set at 80°C for 2 min and subsequently raised to 240°C at a rate of 5°C/min. The temperatures of the injection port and flame ionization detector were maintained at 250°C and 240°C, respectively. Each sample was analyzed independently in triplicate to ensure reproducibility and accuracy of the results.

### 2.9 Statistical analysis

Gene sequencing: The specific steps of 16S rRNA gene sequencing were commissioned by Beijing Qunfeng Nayuan Health Co., LTD. All data were summarized into Microsoft Excel 2019 and statistically analyzed through SPSS version 25.0 software (SPSS Inc., Chicago, IL, United States). The metric data that conformed to the normal distribution were expressed as means ± standard errors (Mean ± SE), and two-tailed independent samples t-test were used between the two groups. Measurements that did not conform to the normal distribution were expressed as median (25th percentile, 75th percentile) [M (P25, P75), and the Wilcoxon test was used between the two groups. Taking α=0.05 as the test level, P<0.05 was statistically significant.

## 3 Results

### 3.1 Basic characteristics of the control group and the case group

A total of 59 cases were enrolled during the study period, including 31 cases in the case group, with an average age of 4.77 (3.20, 8.76) months and a male-to-female ratio of 11:20. Among them, 27 cases were breastfed or predominantly breastfed, and 4 cases were formula-fed or predominantly formula-fed. There were 28 cases in the control group, with an average of 5.50 (3.78, 8.00) months and a male-to-female ratio of 14:14. Among them, 20 cases were breastfed or predominantly breastfed, and 8 cases were formula-fed or predominantly formula-fed. There was no significant difference in gender, age and feeding pattern between the two groups (р > 0.05), as shown in **Table 1**.

**Table 1:** The comparison between control and case group.

Factors		Case group	Control group	*χ2/Z*	*P*
		(n=31)	(n=28)		
Gender	Male	11 (35.5%)	14 (50.0%)	1.270	0.260
	Female	20 (64.5%)	14 (50.0%)		
Age		4.77 (3.20, 8.76)	5.50 (3.78, 8.00)	0.871	0.435
Feeding Pattern	Breastfed or predominantly breastfed	27 (87.1%)	20 (71.4%)	2.229	0.197
	Formula-fed or predominantly formula-fed	4 (12.9%)	8 (28.6%)		

### 3.2 Sample screening and preliminary statistics of sequencing data

**Figure 2:**
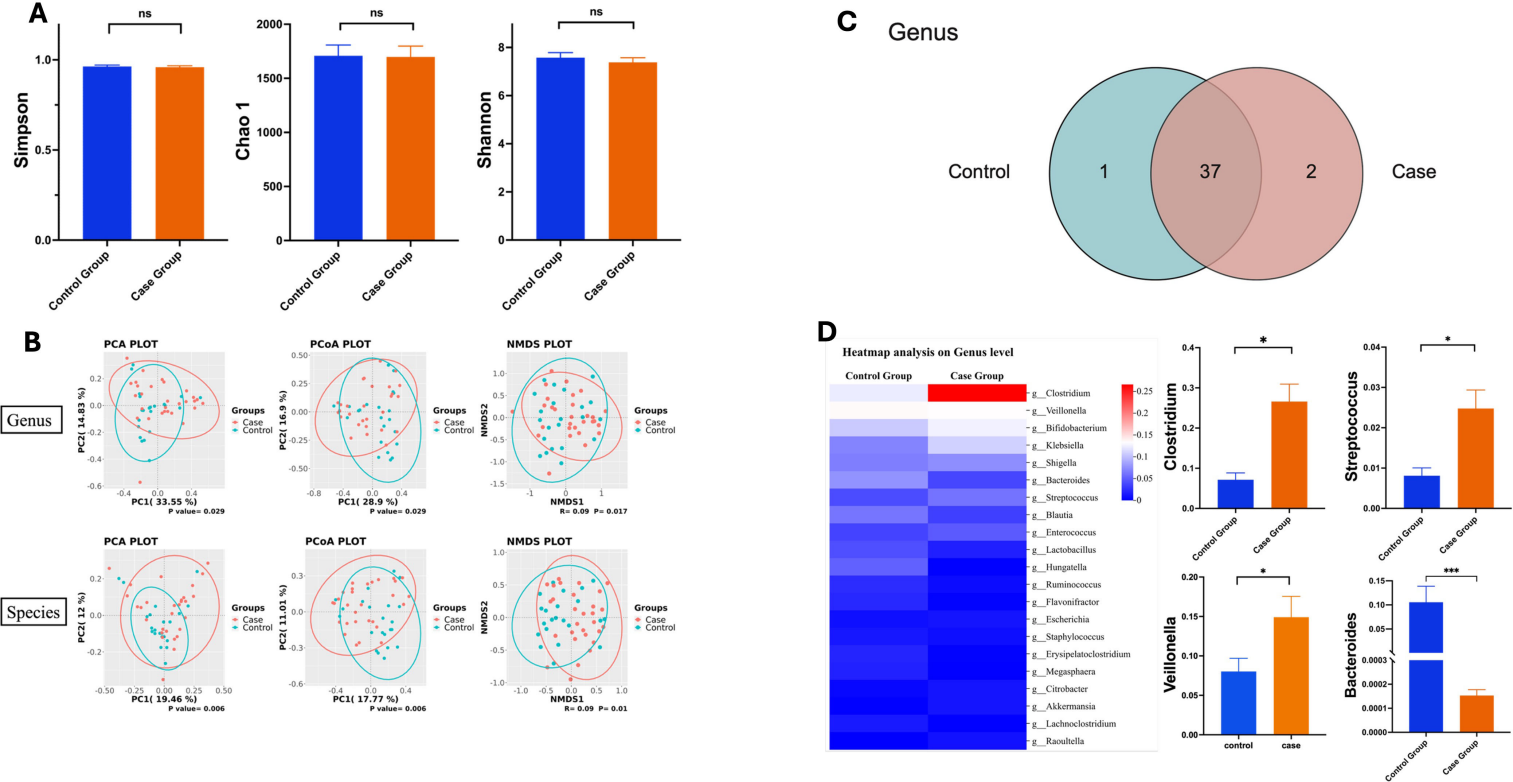
Characteristics and functional alterations in the gut microbiota bewteen two groups. **(A)** a-diversity, **(B)** B-diversity, **(C)** Venn Case Group diagram analysis showing the total number of core species shared and unique, **(D)** Heatmap based on the relative abundance of the general between the two groups (Left), Comparisons of the relative abundances at the genus level according to the 16s rRNA results (Right). *, p<0.05; ns, no significance.

A total of 59 fecal samples were collected and analyzed, including 31 cases in the cases group (marked as M1-M31) and 28 cases in the control group (marked as M41-M43, C41-C65). Samples with extraction or library construction failure were excluded, resulting in a final size of 51, consisting of 29 cases from the case group and 22 from the control group. Meanwhile, A corresponding dilution curve was established using the final 51 samples to reflect whether the sequencing depth was sufficient. It shown that 51 samples tended to level off or reached a plateau phase reflecting an adequate sequencing (**Supplementary Figure S1**). We compared the α-diversity between the control group and case group. No significant difference was found in Shannon, Simpson, and Chao 1, as shown in **Figure 2A**. Moreover, the differences of β-diversity in Genus and Species level between the two groups were analyzed, the upper image represents the Genus level, while the lower one represents the species level. As illustrated in **Figure 2B**, at the genus level, PCA, PCoA, and NMDS revealed that the gut microbiota composition of case group was significantly different from that of control group (p<0.05, PERMANOVA test). Furthermore, our findings at the species level corroborated the results observed at the genus level. This finding indicated that the diversity within both groups was found to be comparable, however, the composition of the microbial communities exhibited significant differences between the two groups. **Figure 2C** presented a Venn diagram illustrating the shared and unique species between the control group and the case group. In total, 37 genera were identified across both groups, with 1 genus being unique to the control group and 2 genera exclusive to the case group.

Furthermore, we performed Operational Taxonomic Unit (OTU) cluster analysis and abundance analysis at the genus, species, and phylum levels (**Supplementary Figures S2a–c**). As depicted in **Supplementary Figure S2d** at the genus level, the top three genera with the highest relative abundance were *Clostridium*, *Veillonella*, and *Bifidobacterium*. Notably, most samples from the case group were clustered on the left side of the diagram, whereas samples from the control group were predominantly clustered on the right (**Supplementary Figure S2b**). At the phylum level, both groups exhibited the highest abundances of *Firmicutes* and *Proteobacteria*, followed by *Bacteroidetes* and *Actinobacteria* (**Supplementary Figure S2d**). At the species level, the three most abundant bacterial species across the two groups were *Clostridium butyricum*, *Bifidobacterium longum*, and *Veillonella parvula* (**Supplementary Figure S2d**). It is important to note that at both the genus and species levels, only a few samples from the same group clustered together on one side, indicating that the abundance distribution was relatively similar between the two groups. **Figure 2D** illustrated that SLI infants showed positively significant difference on the relative abundance of *Clostridium*, *Streptococcus*, *Veillonella* than the control group, while the relative abundance of *Bacterorides* was lower than the control group significantly.

**Figure 3:**
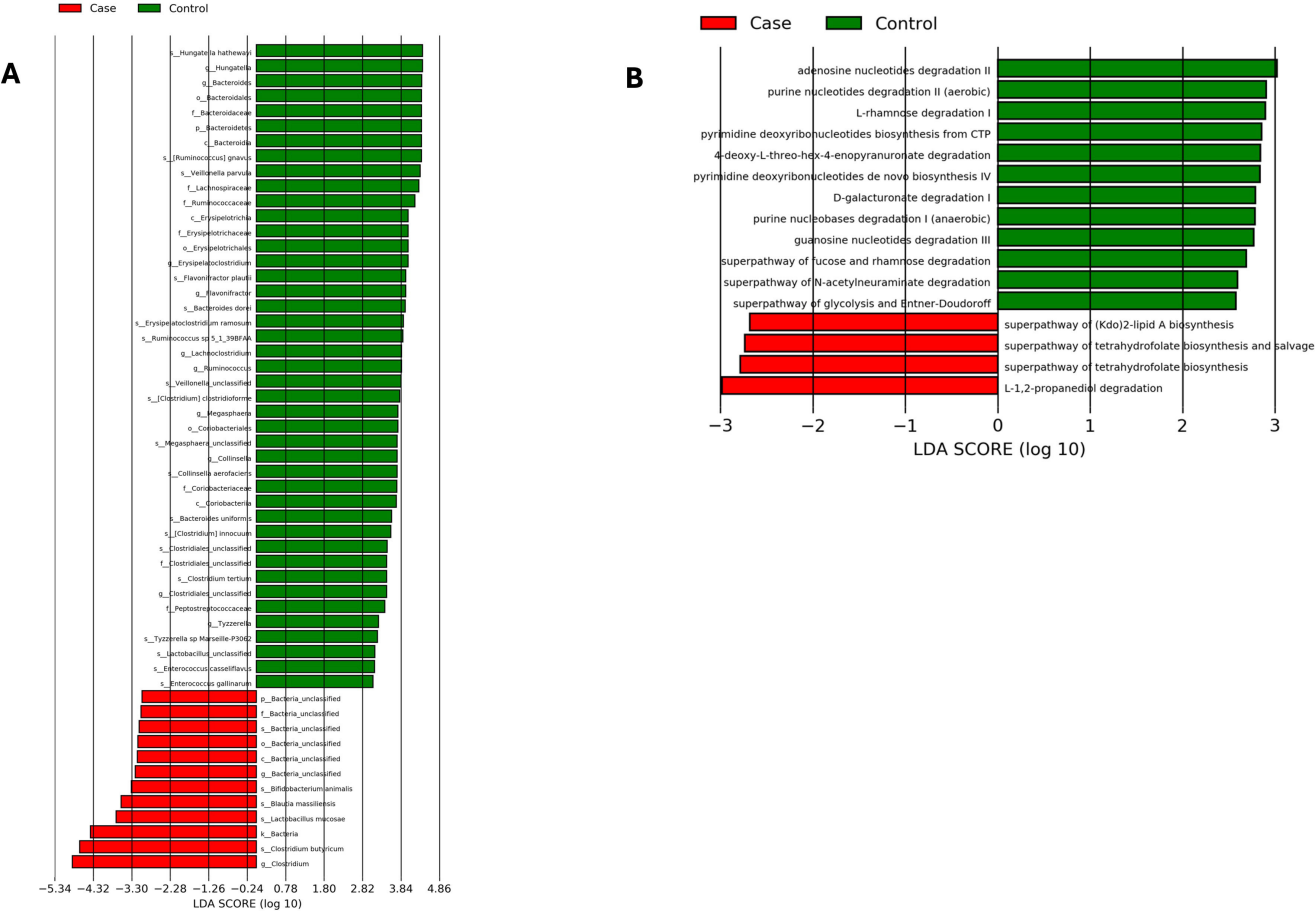
Linear discriminant analysis (LDA) score of the statistically significant microbial groups in the two groups. **(A)** Gut microbiota alterations were assessed by comparing the two groups using LEfSe biomarker discovery tool. **(B)** Predicting differences in metabolic pathways using PICRUSt2 and LEfSe.

Moreover, in order to detect enrichment of specific bacterial taxa among the two groups, we performed linear discriminant analysis (LDA) effects size (LDA score threshold of > 2, p < 0.05), which emphasizes statistical significance and biological consistency. Composition analysis revealed significant alterations in microbial abundance at both the genus and species levels when comparing patients with SLI to control individuals. At the phylum level, the phylum *Bacteroidetes* was significantly reduced in the case group (P<0.05). At genus level, the case group showed a reduced abundance of *Bacteroides*, *Collinsella*, *Ruminococcus*, and *Flavonifractor*, while there was a greater abundance of *Clostridium* (**Figure 3A**). At the species level, the species *Ruminococcus gnavus* and *Veillonella parvula* were significantly reduced in the case group (P<0.05), while the species *Clostridium butyricum*, *Lactobacillus mucosae*, *Blautia* massiliensis, and *Bifidobacterium animalis*, were significantly increased (P<0.05). Interestingly, at the Kingdom level, there was a significant increase in the abundance of bacteria within the case groups. Subsequently, we used Picrust2 to infer microbial community genomic functions between the two groups as shown in **Figure 3B**. It was found that the case group exhibited significantly lower levels of several metabolic pathways compared to the control group. Specifically, the Super-pathway of Glycolysis and Entner-Doudoroff, as well as L-rhamnose Degradation I in the case group, were significantly reduced. Additionally, D-galacturonate Degradation I, and the Super-pathway of Fucose and Rhamnose Degradation were also found to be significantly lower (P < 0.05) in the case group. These results suggest a potential disruption in key metabolic processes within the bacterial community of the case group.

### 3.3 Different comparisons of metabolites in feces between two groups

**Figure 4:**
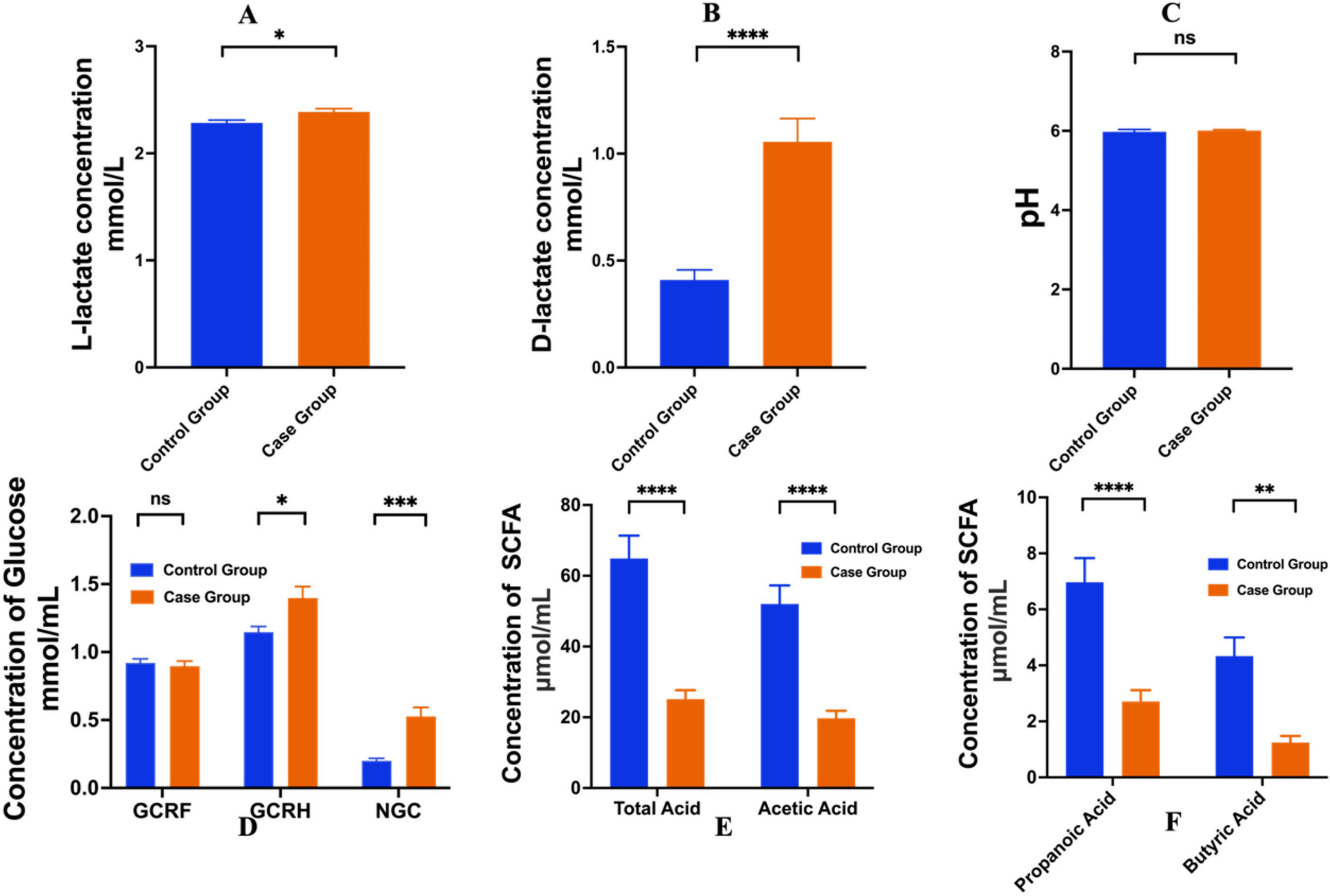
Comparative analysis of pre-fermentation metabolites in feces between Control and Case Group. **(A)** L-lactate content in fecal samples, **(B)** D-lactate content in fecal samples, **(C)** pH values of fecal samples, **(D)** The glucose concentration of GCRF, GCRH and NGC, **(E, F)** SCFA contents in fecal samples including total acid, acetic acid, propanoic acid, and butyric acid. *, p<0.05; **, p<0.01; ***, p<0.001; ****, p<0.0001; ns, no significance.

To demonstrate the differences of metabolites in feces between control group and case group, analysis of L-lactate, D-lactate, pH, glucose concentration, SCFA content was measured as illustrated in **Figures 4A–F**). The analysis indicated there was no significant difference in L-lactate levels between the two groups. In contrast, a significant disparity was noted in D-lactate content, with the case group exhibiting significantly higher levels compared to the control group (p < 0.0001), as depicted in **Figures 4A, B**. Furthermore, the pH values did not reveal a significant variation between the two groups, as depicted in **Figure 4C**. As shown in **Figure 4D**, in terms of NGC, the glucose concentration in the case group was 0.53 mmol/mL, exhibiting a significant difference from the corresponding value in the control group (0.2 mmol/mL). Additionally, Regarding GCRH, the glucose concentration in the case group was recorded at 1.40 mmol/mL, significantly displaying a disparity from the concentration observed in the control group (1.148 mmol/mL). Conversely, the GCRF content between the two groups did not demonstrate statistical significance (P > 0.05). While measurements of fecal concentrations of isobutyric acid, isovaleric acid and valeric acid were taken, the results yielded exceedingly low values, leading to the decision not to further discuss these findings. Notably, **Figures 4E, F** illustrate the varying SCFA content between the control and case groups, with significant differences observed in all SCFAs measured (p < 0.05).

In addition, the ratio of various fatty acids to the total short-chain fatty acid (SCFA) were analyzed and are presented in **Table 2**. Specially, the proportions of acetic acid, propionic acid, and butyric acid in the fecal matter of the case group were approximately 79.11%, 9.54%, 5.74%, respectively. In contrast, the control group exhibited proportions of these three acids at around 79.99%, 10.02%, 5.3%, respectively. Notably, the case group demonstrated lower percentages of propionic acid and butyric acid compared to the control group, while the proportion of acetic acid was higher in the case group. Despite these observations, the differences in the ratios of these fatty acids between the two groups were statistically insignificant (P > 0.05). These results indicate that while there are variations in the composition of SCFAs, they do not reach statistical significance, suggesting that further investigation may be necessary to determine the implications of these findings on gut health and metabolic processes.

**Table 2:** The ratio of acetic acid, propionic acid and butyric acid in total SCFA.

Samples	Control group	Case group	*Z*	*P*
Acetic acid/Total acid (%)	79.99 ± 0.08	79.11 ± 0.10	–	0.70
Propanoic acid/Total acid (%)	10.02 (6.47, 12.81)	9.54 (5.42, 14.54)	-0.41	0.68
Butyric acid/Total acid (%)	5.3 (1.89, 8.51)	5.74 (2.62, 14.05)	-0.56	0.57

### 3.4 Determination of different metabolites after fermentation based on different mediums

**Figure 5:**
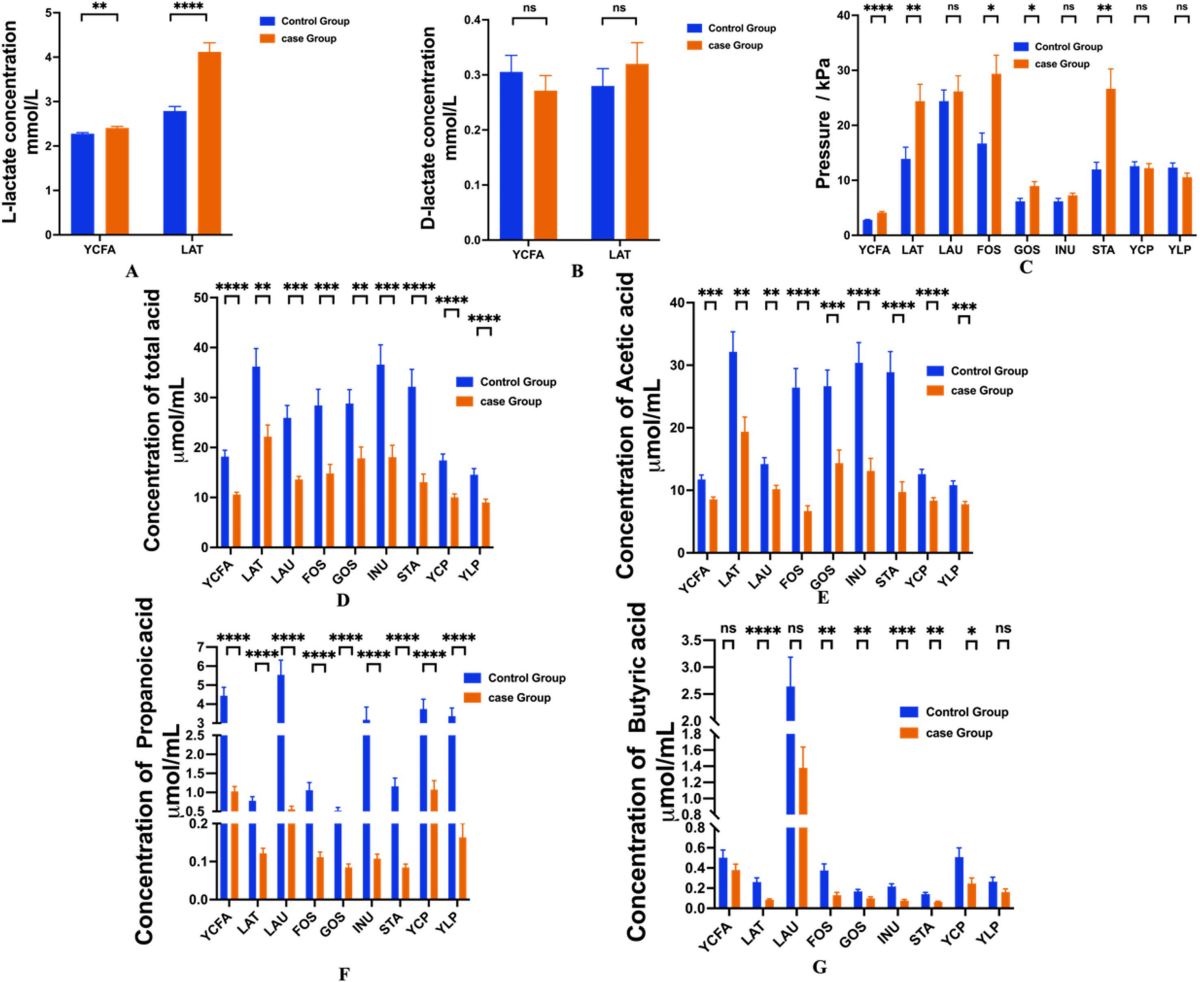
Comparative analysis of metabolites after fermentation in feces between Control and Case Group based on different medium. Data shows in means tsem. **(A)** L-lactose content, **(B)** D-lactate content, **(C)** Gas pressure of fecal samples, **(D-F)** SCFA contents in fecal samples. 40 including total acid **(D)**, acetic acid **(E)**, propanoic acid **(F)**, and butyric acid **(G)**. YCFA, base medium; LAT, lactose medium; LAU, lactulose medium; FOS, fructo-oligosaccharide medium; GOS, galactooligosaccharide medium; INU, inulin medium; STA, soluble starch medium; YCP, hydrolysed casein protein medium; YLP, hydrolyzed whey protein medium level. *, p<s0.05; **, p<0.01; ***, p<0.001; ****, p<0.0001; ns, no significance.

To compare the metabolic activity of gut microbiota from LI infants and healthy infants under various substrate conditions and to prepare for future intervention strategies for LI infants, 10% infant fecal suspension was cultured in various oligosaccharides and hydrolyzed protein mediums for *in vitro* fermentation, and gas production and short-chain fatty acid profiles were subsequently measured after 24-hour fermentation in different media. The results were illustrated in **Figures 5A–G**.

It was found that the concentration of L-lactate in the case group in LAT medium was significantly higher compared to the control group (p<0.0001), which was consistent with the results in YCFA medium (**Figure 5A**). **Figure 5B** shows that in the LAT medium, the case group exhibited higher levels of D-lactate relative to the control group; however, in the YCFA medium, the D-lactate content was lower in the case group compared to controls. Despite these findings, the differences between the two groups in both YCFA and LAT media did not reach statistical significance. The gas pressure presented in **Figure 5C** revealed that the infant feces from the case group produced significantly more gas in the LAT medium than those from the control group (p < 0.05), after 24 hours of *in vitro* anaerobic fermentation. This outcome was consistent with the gas production results in the YCFA, FOS, GOS, and STA media (p < 0.05). Conversely, no statistically significant differences were found in gas production for LAU, INU, YCP, and YLP media (p > 0.05).

**Figures 5D–G** demonstrated the SCFA content post-fermentation, including total acid, acetic acid, propionic acid, and butyric acid. Following 24 hours of *in vitro* fermentation, it was found that the infant feces from the case group exhibited significantly lower levels of total acid, acetic acid, and propionic acid in the LAT medium compared to those from the control group (p < 0.05). Similar patterns in the concentrations of total acid, acetic acid, and propionic acid were observed in the other media analyzed (**Figures 5D–F**). **Figure 5G** highlights that the concentration of butyric acid in the case group was significantly lower than that in the control group regarding LAT media, a finding consistent with analyses conducted in the FOS, GOS, INU, STA, and YCP media. On the contrary, no significant differences were noted between the two groups in the YCFA, LAU, and YLP media. Collectively, these results suggest distinct differences in metabolic fermentation profiles and SCFA production between the case and control groups, indicating potential implications for gut health in infants.

**Figure 6:**
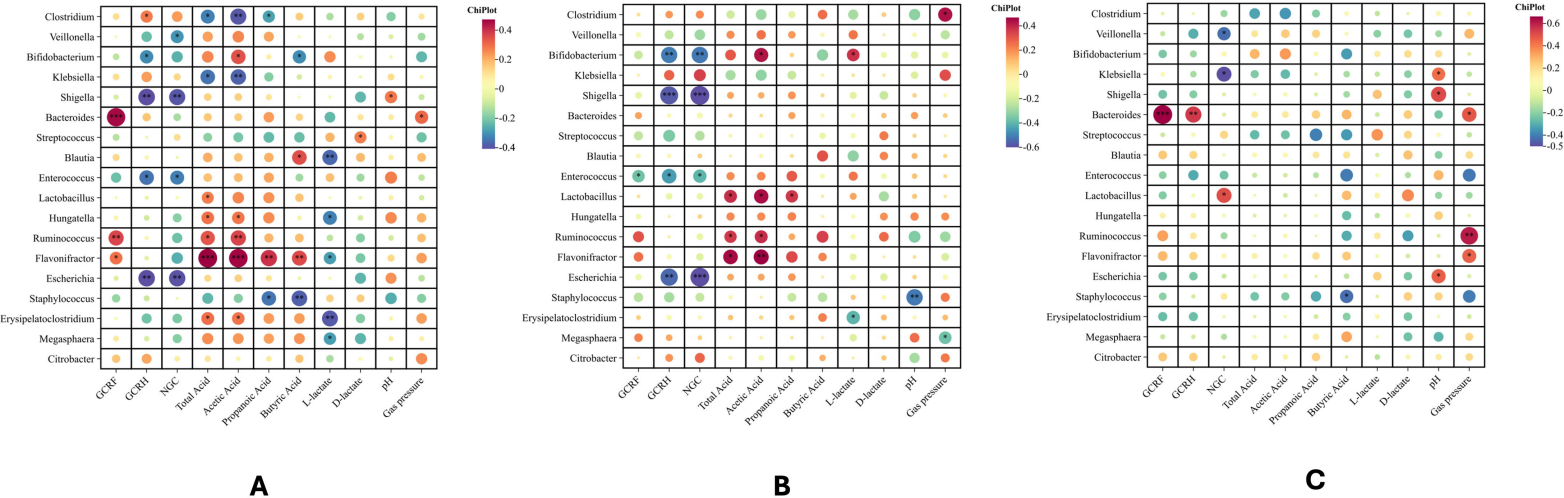
Spearman's correlation between identified taxa and detected fecal microbiota on genus group only. **(A)** Spearman's correlation containing Case and Control group, **(B)** Case group only, **(C)** Control. *, p<0.05; **, p<0.01;***,p<0.001.

To further understand the relationship between identified taxa and fecal microbiota on genus level, a Spearman’s correlation was performed that included all samples in the case coup & control group (**Figure 6A**), only in the case group (**Figure 6B**), only in the control group (**Figure 6C**). It revealed that, in the combined group (control + case group), certain taxa such as *Clostridium* and *Klebsiella* were significantly negatively correlated with several SCFAs, including acetate and propionate, whereas such genera like *Bifidobacterium*, *Ruminococcus*, and *Flavonifractor* and showed positive associations. When stratified by group, correlations in the case group were notably stronger and more clustered, suggesting a disturbed microbial-metabolite network. *Clostridium* showed a positive association with gas pressure. *Bifidobacterium*, *Lactobacillus*, *Ruminococcus*, and *Flavonifractor* showed positive associations with total acids, acetic acid, propanoic acid. Notably, there was a positive association between *Bifidobacterium* and L-lactate. In contrast, the control group exhibited fewer and weaker correlations, implying a more balanced and functionally stable gut microbiota. Notably, *Bifidobacterium* and *Lactobacillus* showed significant positive correlations with L-lactate, acetate and propionate respectively.

## 4 Discussion

In contrast to primary lactose intolerance, which is primarily driven by genetically regulated downregulation of lactase expression, SLI in infants is closely associated with intestinal mucosal injury and the delayed maturation of digestive function and gut microbiota. In this specific population, establishing age-appropriate and non-invasive markers is particularly important for elucidating the underlying pathophysiological mechanisms. However, as previously mentioned in the introduction, current diagnostic methods for assessing secondary lactose intolerance in infants have limitations in terms of feasibility, specificity, or applicability. In contrast, fecal residual lactose directly reflects the level of lactose that has neither been absorbed in the small intestine nor further utilized by the gut microbiota, providing a more direct phenotypic indicator for evaluating abnormal lactose digestion and fermentation under conditions of intestinal dysfunction. Based on this rationale, to systematically characterize the lactose metabolism and associated gut microbiological alterations in infants with secondary lactose intolerance, this study explored fecal residual lactose as a non-invasive functional marker for impaired lactose digestion and utilization. We conducted a comprehensive analysis integrating *in vitro* fecal fermentation assays and 16S rRNA gene sequencing to compare the gut microbial composition and metabolic profiles between Chinese infants with lactose intolerance and healthy controls. To the best of our knowledge, this study provides a relatively comprehensive characterization of gut microbiota diversity and lactose metabolic features in a Chinese infant cohort with SLI, thereby offering valuable insights into the microbial and metabolic characteristics linked to SLI during early life.

In the diet of normal infants, most ingested lactose is effectively digested and absorbed by LPH located on the brush border of the small intestine. When the quantity of lactose consumed surpasses the threshold that the small intestine can effectively digest and absorb, or in cases where small intestinal diseases lead to mucosal damage, there can be a reduction in LPH secretion or activity. As a result, the undigested lactose may transit through the digestive tract and reach the colon due to intestinal peristalsis. Previous literature indicated that regardless of the feeding method, approximately 20% of lactose can escape digestion and enter the colon for fermentation in normal infants (24, 25). In the present study, lactose residues were likewise detected in the feces of healthy infants, supporting the notion that partial lactose escape from the digestion by LPH in the small intestine. This observation aligns with findings from both domestic and international studies (26). In contrast, the residual lactose content in the feces of case group was approximately double that of their normal counterparts. This observation suggests a reduction or absence of lactase enzymes on the brush border of small intestinal epithelial cells, resulting in elevated levels of lactose entering the colon.

When lactose enters the colon, it is first hydrolyzed into glucose and galactose by lactase-expressing bacteria such as *Lactobacillus*, *Bacteroides*, and *Bifidobacterium.* Galactose is subsequently converted into glucose via the Leloir pathway. These monosaccharides are further metabolized by the gut microbiota to generate pyruvate, which is primarily converted into acetyl-CoA. At the same time, Lactic acid, succinic acid, H_2_, CO_2_ and other gases can be produced (14, 27), which can be further fermented into Short-chain fatty acid (SCFA), including acetic acid, propionic acid, butyric acid, and CH_4_. Lactic acid is an intermediate metabolite of SCFAs, which includes both L-lactic acid and D-lactic acid (28). Under normal physiological conditions, the human body produces limited amounts of D-lactic acid. An appropriate concentration of lactic acid is beneficial for enhancing the integrity of the intestinal barrier and plays a crucial role in regulating intestinal immune function (28, 29). In this study, fecal metabolite measurements represent the integrated *in vivo* outcome of lactose digestion, microbial fermentation, host absorption, and intestinal transit. A general reduction in total SCFA levels, including acetate, propionate, and butyrate, was observed in the case group. Moreover, the levels of D-lactic acid and L-lactic acid were significantly higher than those of the control group, suggesting that the process of lactose fermentation may shift towards incomplete fermentation. In addition, Spearman’s correlations also demonstrated that the L-lactate content was positively significant with the abundance of *Bifidobacterium* in **Figure 6B**, which is consistent with previous reports that *Bacteroides*, *Lactobacillus*, and *Bifidobacterium* were able to produce lactic acid (30, 31). The above metabolic results can be partially explained by the reduced abundance of key acetic and propionic acid-producing taxa, such as *Ruminococcus* and *Bacteroidetes*. It should be emphasized that fecal SCFA levels reflect the balance among microbial production, host absorption, and colonic metabolic processes (32). Therefore, the decrease in SCFA should not be attributed only to the decrease in the abundance of SCFA-producing bacteria. In addition to altered microbial composition, host absorption of SCFA, changes in colonic pH, and competitive utilization of fermentation substrates between SCFA synthesis and gas production pathways may all collectively shape the final metabolite profile (32, 33). In the present study, although fecal lactate levels were elevated, fecal pH remained unchanged between groups, arguing against a dominant role of pH-driven effects. Moreover, given the context of intestinal mucosal impairment and altered microbial community structure in secondary lactose intolerance, enhanced SCFA absorption is unlikely to be the primary determinant (13, 34). The observed metabolite profiles are likely shaped by a combination of microbial and host-related factors. A more plausible explanation is that impaired lactose digestion due to reduced LPH activity in infants with SLI may increase the delivery of unabsorbed lactose to the colon, thereby altering substrate availability for microbial fermentation. This host-driven malabsorption may secondarily affect microbial metabolic pathways in favor of lactate accumulation and modified SCFA production.

To further explore microbial metabolic characteristics under controlled conditions, we performed *in vitro* fecal fermentation experiments, which were designed to assess the potential metabolic capacity of the fecal microbiota when exposed to standardized substrates. It should be emphasized that these *in vitro* systems do not aim to fully recapitulate the infant colonic environment, particularly with respect to substrate availability, intestinal transit time, host absorption, and host–microbe interactions. Rather, they provide a comparative framework to evaluate group-specific differences in microbial metabolic responses under identical fermentation conditions. Consistent with the fecal metabolite data, results from *in vitro* fecal fermentation indicated that the concentration of L-lactic acid in the case group was significantly elevated compared to the normal group when cultured in the YCFA and LAT media. This indicates a metabolic shift in the gut microbiota of case group toward a lactate-producing pathway when utilizing carbohydrate substrates such as lactose. Additionally, He et al. (2) reported that in the presence of lactose, although lactose hydrolysis efficiency did not differ significantly between the lactose-tolerant and intolerant groups, the intolerant group fecal bacteria showed faster lactate and SCFA production rates in the early fermentation stage, suggesting group-specific differences in colonic lactose metabolism patterns.

There are two ways for colonic flora to ferment lactose, one is the non-gas producing way, and the other is the gas producing way. In the gas-producing pathway, the colonic flora ferments lactose, generating substantial amounts of H_2_, CO_2_, and CH_4_, most of which are typically absorbed in the intestinal lumen. However, when the production of these gases surpasses the absorption capacity of the intestinal cavity, symptoms such as abdominal distension, bowel discomfort, and excessive flatulence may arise (35, 36). In addition to lactose, colonic bacteria are also capable of metabolizing other polysaccharides such as FOS, GOS, inulin, and soluble starch. Protein metabolism in the colon is also of increasing interest. As an essential dietary component, protein supports infant growth and development, facilitates muscle and bone metabolism, and is crucial for the maintenance and maturation of the nervous system (37). Given the widespread incorporation of oligosaccharide-based prebiotics (e.g., FOS and GOS) in infant formula, it is important to evaluate the metabolic profiles of infant gut microbiota under different substrate conditions. Our results demonstrated a significant reduction in total acid, acetate, and propionate levels in the case group across all culture media, suggesting a general impairment in glycolysis and SCFA production capacity. This may be attributed to a decrease in key acid-producing bacteria (e.g., acetate- and propionate-producing species) or diminished metabolic activity, reflecting a disrupted microbial cross-feeding network.

Notably, butyrate levels did not significantly decrease under basic, LAU, or casein hydrolysate culture conditions, indicating that butyrate-producing capacity may be partially preserved under specific substrates. However, in fermentation systems rich in fermentable carbohydrates—such as GOS, FOS, and starch—butyrate production was significantly lower in the case group. This suggests a possible shift in microbial composition, where certain taxa may preferentially utilize these carbon sources, potentially suppressing the growth or metabolic activity of butyrate-producing bacteria (38). Furthermore, gas production in the case group was significantly higher under multiple carbon-rich conditions (e.g., LAT, GOS, FOS, and soluble starch), indicating excessive fermentation activity. This may be related to malabsorption of lactose and other carbohydrates, allowing more substrates to reach the colon and drive gas production. This increased gas production likely represents a microbial metabolic phenotype associated with lactose malabsorption, rather than a direct measure of clinical symptom severity. Members of the *Clostridium* genus, known producers of hydrogen (H_2_) and carbon dioxide (CO_2_) (28, 29), were significantly more abundant in the case group, as mentioned in **Figure 6B**, *Clostridium* showed a positive association with gas pressure. Importantly, due to the absence of standardized clinical symptom assessment or stool characterization, the relationship between microbial gas production and infant symptoms cannot be directly evaluated in this study. Therefore, increased gas production could represent a microbial metabolic response associated with carbohydrate malabsorption in the case group.

We also performed 16S rRNA gene sequencing of fecal samples from both groups. Compared with the adult gut microbiota, the infant gut microbiota is generally characterized by lower diversity, structural instability, and high variability. In the present study, the dominant phyla in both groups were *Firmicutes* and *Proteobacteria*, followed by *Bacteroidetes* and *Actinobacteria*, consistent with previous reports (39). However, we observed marked compositional differences between groups. In parallel with the decrease in *Bacteroidetes*, sevel key *Firmicutes*-associated taxa-such as the class *Erysipelotrichia*, families *Lachnospiraceae* and *Ruminococcaceae*, as well as the genera *Megasphaera*, and species *Veillonella parvula*-were also reduced relative to controls. Additionally, β-diversity analysis revealed significant separation between the two groups, indicating a structural dysbiosis of gut microbiota among case group. Functionally, members of the *Bacteroides* genus primarily ferment carbohydrates via the succinate-propionate pathway (40) generating CO_2_, small amounts of H_2_, and propionate, or via the pyruvate-acetate pathway (41). *Ruminococcus*, particularly *Ruminococcus bromii*, plays a pivotal role in resistant starch fermentation and shapes butyrate production in the gut through metabolic cross-feeding with established butyrate-producing taxa, thereby supporting intestinal energy homeostasis and epithelial integrity (42). Certain *Clostridium* species can convert lactate and acetate into butyrate (43) and members of the genus *Veillonella* are known to convert lactate into propionate via the succinate-propionate pathway, thereby contributing to propionate production in the gut (44).

Overall, this study characterizes a set of microbial and metabolic features associated with secondary lactose intolerance in a cohort of Chinese infants. The results indicated that the case group exhibited accumulation of lactate and decrease in SCFA more consistent with the combined effects of host-driven lactose malabsorption and microbial metabolic responses rather than being driven by a single pathogenic factor. Importantly, dietary exposures, feeding practices, and early-life microbial colonization patterns vary substantially across regions and populations, and these factors are likely to influence the specific microbial taxa involved and the magnitude of metabolic alterations observed (45). Infant feeding mode, including breastfeeding and formula feeding, has been shown to profoundly shape gut microbiota composition and functional capacity during early life, with downstream effects on carbohydrate fermentation and metabolite production (46). Therefore, the present findings should not be interpreted as directly generalizable to all infant populations, but rather as providing a mechanistic reference for understanding how secondary lactose intolerance may manifest at the microbiota–metabolism interface under specific dietary and environmental contexts.

## 5 Limitations

This study acknowledges several limitations that should be considered when interpreting the results. Firstly, the analysis was based on 16S rRNA rather metagenomic sequencing, which restricts our ability to conduct a more comprehensive analysis of the microbial communities present in the gut. In addition, the absence of blood-based measurements limit our ability to assess systemic metabolic or inflammatory responses associated with lactose intolerance and gut microbiota alterations. Integrating blood analysis with metagenomic sequencing in future studies could yield more detailed insights into the physiological responses and metabolic profiles associated with lactose intolerance. Secondly, fecal metabolite profiles and gas production were assessed using a combination of *in vivo* measurements and controlled *in vitro* fermentation assays. While these approaches provide complementary insights into microbial metabolic outcomes and potential, the *in vitro* systems cannot fully replicate the complexity of the infant colonic environment, including substrate availability, intestinal transit, gas absorption, and host–microbe interactions. Future studies incorporating longitudinal clinical symptom scoring, stool profiling, and integrated microbiome–metabolome analyses will be necessary to further clarify the clinical relevance of microbial gas production in infant lactose intolerance.

Lastly, this study was conducted in a cohort of Chinese infants with a relatively limited sample size. Dietary exposures, feeding practices, and early-life microbial colonization patterns vary across populations. Therefore, we recommend that future experiments involve larger cohorts to enhance the statistical power and generalizability of the findings. A more extensive participant pool would allow for a better understanding of the variations in gut microbiota and lactose metabolism across different populations of infants. Addressing these limitations in future research will be essential to further elucidate the complex interactions between gut microbiota, lactose metabolism, and infant health.

## 6 Conclusion

Despite the limited sample size, our study highlights the potential utility of residual fecal lactose as a non-invasive indicator associated with SLI in infants. In this cohort of Chinese infants, SLI exhibited elevated levels of lactate, increased gas production, and distinct gut microbiota dysbiosis, characterized by reduced SCFA production. These alterations are consistent with a possible pathophysiological mechanism whereby epithelial damage in the small intestine is associated with decreased LPH secretion, impaired lactose digestion, and malabsorption. Unabsorbed lactose is partially excreted contributing to an increased fecal lactose content, and partially fermented by a dysbiotic microbiota with reduced metabolic efficiency, resulting in lactate and gas accumulation. When these fermentation byproducts exceed the colonic reabsorption capacity, they may increase luminal osmotic pressure and are pathophysiologically consistent with clinical features commonly observed in lactose malabsorption, such as osmotic diarrhea and abdominal distension. Further studies with expanded cohorts are warranted to validate these findings. In particular, qualitative profiling of fermentation gases and integrative analysis using whole-genome metagenomic sequencing will be essential to clarify the functional role of gut microbiota in the onset and progression of SLI, ultimately guiding future diagnostic and therapeutic strategies.

## Data Availability

The datasets generated for this study can be found in the NCBI SRA repository under the accession number PRJNA1277478; https://www.ncbi.nlm.nih.gov.

## Supplementary material

Supplementary Figure 1 The dilution curves of the samples.

Supplementary Figure 2 The OTU heatmap of the relative abundance in two groups **(a–c)**, **(d)** Relative abundance distribution of two groups.

## References

[1] Montgomery RK Krasinski SD Hirschhorn JN Grand RJ . Lactose and lactase--who is lactose intolerant and why? J Pediatr Gastroenterol Nutr. (2007) 45 Suppl 2:S131–137. doi: 10.1097/MPG.0b013e31812e68f6 18185074

[2] He T Priebe MG Harmsen HJM Stellaard F Sun X Welling GW . Colonic fermentation may play a role in lactose intolerance in humans. J Nutr. (2006) 136:58–63. doi: 10.1093/jn/136.1.58, PMID: 16365059

[3] Szilagyi A Ishayek N . Lactose intolerance, dairy avoidance, and treatment options. Nutrients. (2018) 10:1994. doi: 10.3390/nu10121994, PMID: 30558337 PMC6316316

[4] Anguita-Ruiz A Aguilera CM Gil Á . Genetics of lactose intolerance: an updated review and online interactive world maps of phenotype and genotype frequencies. Nutrients. (2020) 12:2689. doi: 10.3390/nu12092689 32899182 PMC7551416

[5] Thiagarajah JR Kamin DS Acra S Goldsmith JD Roland JT Lencer WI . Advances in evaluation of chronic diarrhea in infants. Gastroenterology. (2018) 154:2045–2059.e6. doi: 10.1053/j.gastro.2018.03.067 29654747 PMC6044208

[6] Shepherd SJ Lomer MCE Gibson PR . Short-chain carbohydrates and functional gastrointestinal disorders. Am J Gastroenterol. (2013) 108:707–17. doi: 10.1038/ajg.2013.96 23588241

[7] Catanzaro R Sciuto M Marotta F . Lactose intolerance—Old and new knowledge on pathophysiological mechanisms, diagnosis, and treatment. SN Compr Clin Med. (2021) 3:499–509. doi: 10.1007/s42399-021-00792-9, PMID: 30311153

[8] Suchy FJ Brannon PM Carpenter TO Fernandez JR Gilsanz V Gould JB . National Institutes of Health Consensus Development Conference: lactose intolerance and health. Ann Intern Med. (2010) 152:792–6. doi: 10.7326/0003-4819-152-12-201006150-00248, PMID: 20404261

[9] Santonocito C Scapaticci M Guarino D Annicchiarico EB Lisci R Penitente R . Lactose intolerance genetic testing: is it useful as routine screening? Results on 1426 south-central Italy patients. Clin Chim Acta. (2015) 439:14–7. doi: 10.1016/j.cca.2014.09.026, PMID: 25281930

[10] Ruzsanyi V Heinz-Erian P Entenmann A Karall D Müller T Schimkowitsch A . Diagnosing lactose malabsorption in children: difficulties in interpreting hydrogen breath test results. J Breath Res. (2016) 10:16015. doi: 10.1088/1752-7155/10/1/016015, PMID: 26934035

[11] Domínguez-Jiménez JL Fernández-Suárez A Ruiz-Tajuelos S Puente-Gutiérrez JJ Cerezo-Ruiz A . Lactose tolerance test shortened to 30 minutes: An exploratory study of its feasibility and impact. Rev Esp Enferm Dig. (2014) 106:381–5. 25361448

[12] Mattar R Basile-Filho A Kemp R dos Santos JS . Comparison of Quick Lactose Intolerance Test in duodenal biopsies of dyspeptic patients with single nucleotide polymorphism LCT-13910C>T associated with primary hypolactasia/lactase-persistence. Acta Cir Bras. (2013) 28 Suppl 1:77–82. doi: 10.1590/s0102-86502013001300015, PMID: 23381829

[13] Heyman MB . Committee on Nutrition. Lactose intolerance in infants, children, and adolescents. Pediatrics. (2006) 118:1279–86. doi: 10.1542/peds.2006-1721, PMID: 16951027

[14] Misselwitz B Butter M Verbeke K Fox MR . Update on lactose malabsorption and intolerance: pathogenesis, diagnosis and clinical management. Gut. (2019) 68:2080–91. doi: 10.1136/gutjnl-2019-318404, PMID: 31427404 PMC6839734

[15] He T Priebe MG Zhong Y Huang C Harmsen HJM Raangs GC . Effects of yogurt and bifidobacteria supplementation on the colonic microbiota in lactose-intolerant subjects. J Appl Microbiol. (2008) 104:595–604. doi: 10.1111/j.1365-2672.2007.03579.x, PMID: 17927751

[16] Zhong Y Priebe MG Vonk RJ Huang C-Y Antoine J-M He T . The role of colonic microbiota in lactose intolerance. Dig Dis Sci. (2004) 49:78–83. doi: 10.1023/b:ddas.0000011606.96795.40, PMID: 14992439

[17] Fukuda S Toh H Hase K Oshima K Nakanishi Y Yoshimura K . Bifidobacteria can protect from enteropathogenic infection through production of acetate. Nature. (2011) 469:543–7. doi: 10.1038/nature09646, PMID: 21270894

[18] Arrieta M-C Stiemsma LT Amenyogbe N Brown EM Finlay B . The intestinal microbiome in early life: health and disease. Front Immunol. (2014) 5:427. doi: 10.3389/fimmu.2014.00427, PMID: 25250028 PMC4155789

[19] Group WMGRS de Onis M . WHO Child Growth Standards based on length/height, weight and age. Acta Paediatrica. (2006) 95:76–85. doi: 10.1111/j.1651-2227.2006.tb02378.x, PMID: 16817681

[20] Shi C O’Donoghue M Yang L Tsang H Chen J Zou J . Factors associated with hand washing effectiveness: an institution-based observational study. Antimicrob Resist Infect Control. (2023) 12:85. doi: 10.1186/s13756-023-01293-1, PMID: 37649107 PMC10469426

[21] Bi Y Tu Y Zhang N Wang S Zhang F Suen G . Multiomics analysis reveals the presence of a microbiome in the gut of fetal lambs. Gut. (2021) 70:853–64. doi: 10.1136/gutjnl-2020-320951, PMID: 33589511 PMC8040156

[22] Qin H Jiao J AD Hua M Han K Du H Wang Z . Single-molecule approach to 16S rRNA for vaginal microbiome signatures in response to metronidazole treatment. Microbiol Spectr. (2023) 11:e0170622. doi: 10.1128/spectrum.01706-22, PMID: 37199621 PMC10269914

[23] Wu L Ye S Deng X Fu Z Li J Yang C . Conjugated linoleic acid ameliorates high fat-induced insulin resistance via regulating gut microbiota-host metabolic and immunomodulatory interactions. Nutrients. (2024) 16:1133. doi: 10.3390/nu16081133, PMID: 38674824 PMC11053735

[24] Wang Y Harvey CB Hollox EJ Phillips AD Poulter M Clay P . The genetically programmed down-regulation of lactase in children. Gastroenterology. (1998) 114:1230–6. doi: 10.1016/s0016-5085(98)70429-9, PMID: 9609760

[25] van de Heijning BJM Berton A Bouritius H Goulet O . GI symptoms in infants are a potential target for fermented infant milk formulae: a review. Nutrients. (2014) 6:3942–67. doi: 10.3390/nu6093942, PMID: 25255831 PMC4179197

[26] Romero-Velarde E Delgado-Franco D García-Gutiérrez M Gurrola-Díaz C Larrosa-Haro A Montijo-Barrios E . The importance of lactose in the human diet: outcomes of a mexican consensus meeting. Nutrients. (2019) 11:2737. doi: 10.3390/nu11112737, PMID: 31718111 PMC6893676

[27] Bayless TM Brown E Paige DM . Lactase non-persistence and lactose intolerance. Curr Gastroenterol Rep. (2017) 19:23. doi: 10.1007/s11894-017-0558-9, PMID: 28421381

[28] Todesco T Rao AV Bosello O Jenkins DJ . Propionate lowers blood glucose and alters lipid metabolism in healthy subjects. Am J Clin Nutr. (1991) 54:860–5. doi: 10.1093/ajcn/54.5.860, PMID: 1951157

[29] Louis P Flint HJ . Formation of propionate and butyrate by the human colonic microbiota. Environ Microbiol. (2017) 19:29–41. doi: 10.1111/1462-2920.13589, PMID: 27928878

[30] Gill RK Saksena S Alrefai WA Sarwar Z Goldstein JL Carroll RE . Expression and membrane localization of MCT isoforms along the length of the human intestine. Am J Physiol Cell Physiol. (2005) 289:C846–852. doi: 10.1152/ajpcell.00112.2005, PMID: 15901598

[31] Tsukuda N Yahagi K Hara T Watanabe Y Matsumoto H Mori H . Key bacterial taxa and metabolic pathways affecting gut short-chain fatty acid profiles in early life. ISME J. (2021) 15:2574–90. doi: 10.1038/s41396-021-00937-7, PMID: 33723382 PMC8397723

[32] den Besten G van Eunen K Groen AK Venema K Reijngoud D-J Bakker BM . The role of short-chain fatty acids in the interplay between diet, gut microbiota, and host energy metabolism. J Lipid Res. (2013) 54:2325–40. doi: 10.1194/jlr.R036012, PMID: 23821742 PMC3735932

[33] Walker AW Duncan SH McWilliam Leitch EC Child MW Flint HJ . pH and peptide supply can radically alter bacterial populations and short-chain fatty acid ratios within microbial communities from the human colon. Appl Environ Microbiol. (2005) 71:3692–700. doi: 10.1128/AEM.71.7.3692-3700.2005, PMID: 16000778 PMC1169066

[34] Heine RG AlRefaee F BaChina P De Leon JC Geng L Gong S . Lactose intolerance and gastrointestinal cow’s milk allergy in infants and children – common misconceptions revisited. World Allergy Organ J. (2017) 10:41. doi: 10.1186/s40413-017-0173-0, PMID: 29270244 PMC5726035

[35] Manichanh C Rigottier-Gois L Bonnaud E Gloux K Pelletier E Frangeul L . Reduced diversity of faecal microbiota in Crohn’s disease revealed by a metagenomic approach. Gut. (2006) 55:205–11. doi: 10.1136/gut.2005.073817, PMID: 16188921 PMC1856500

[36] Forsgård RA . Lactose digestion in humans: intestinal lactase appears to be constitutive whereas the colonic microbiome is adaptable. Am J Clin Nutr. (2019) 110:273–9. doi: 10.1093/ajcn/nqz104, PMID: 31175813 PMC6669050

[37] Kårlund A Gómez-Gallego C Turpeinen AM Palo-Oja O-M El-Nezami H Kolehmainen M . Protein supplements and their relation with nutrition, microbiota composition and health: is more protein always better for sportspeople? Nutrients. (2019) 11:829. doi: 10.3390/nu11040829, PMID: 31013719 PMC6521232

[38] Roupar D Coelho MC Gonçalves DA Silva SP Coelho E Silva S . Evaluation of microbial-fructo-oligosaccharides metabolism by human gut microbiota fermentation as compared to commercial inulin-derived oligosaccharides. Foods. (2022) 11:954. doi: 10.3390/foods11070954, PMID: 35407041 PMC8997964

[39] Xiao L Wang J Zheng J Li X Zhao F . Deterministic transition of enterotypes shapes the infant gut microbiome at an early age. Genome Biol. (2021) 22:243. doi: 10.1186/s13059-021-02463-3, PMID: 34429130 PMC8383385

[40] Reichardt N Duncan SH Young P Belenguer A McWilliam Leitch C Scott KP . Phylogenetic distribution of three pathways for propionate production within the human gut microbiota. ISME J. (2014) 8:1323–35. doi: 10.1038/ismej.2014.14, PMID: 24553467 PMC4030238

[41] Miller TL . The pathway of formation of acetate and succinate from pyruvate by Bacteroides succinogenes. Arch Microbiol. (1978) 117:145–52. doi: 10.1007/BF00402302, PMID: 678020

[42] Ze X Duncan SH Louis P Flint HJ . Ruminococcus bromii is a keystone species for the degradation of resistant starch in the human colon. ISME J. (2012) 6:1535–43. doi: 10.1038/ismej.2012.4, PMID: 22343308 PMC3400402

[43] Detman A Mielecki D Chojnacka A Salamon A Błaszczyk MK Sikora A . Cell factories converting lactate and acetate to butyrate: Clostridium butyricum and microbial communities from dark fermentation bioreactors. Microb Cell Fact. (2019) 18:36. doi: 10.1186/s12934-019-1085-1, PMID: 30760264 PMC6373154

[44] Fusco W Lorenzo MB Cintoni M Porcari S Rinninella E Kaitsas F . Short-chain fatty-acid-producing bacteria: key components of the human gut microbiota. Nutrients. (2023) 15:2211. doi: 10.3390/nu15092211, PMID: 37432351 PMC10180739

[45] Zeng S Patangia D Almeida A Zhou Z Mu D Paul Ross R . A compendium of 32,277 metagenome-assembled genomes and over 80 million genes from the early-life human gut microbiome. Nat Commun. (2022) 13:5139. doi: 10.1038/s41467-022-32805-z, PMID: 36050292 PMC9437082

[46] Penders J Thijs C Vink C Stelma FF Snijders B Kummeling I . Factors influencing the composition of the intestinal microbiota in early infancy. Pediatrics. (2006) 118:511–21. doi: 10.1542/peds.2005-282 16882802

