# Supplementary material for: Diagnostic and metabolic insights into secondary lactose intolerance in infants via fecal lactose quantification and gut microbiome profiling
Source: Front Immunol. 2026 Apr 21;17:1711945. doi: 10.3389/fimmu.2026.1711945 (PMC13139010; doi:10.3389/fimmu.2026.1711945)
Supplement: Supplementary Figure 1 — The dilution curves of the samples. [file DataSheet1.zip › Data Sheet 1/figure_supplymentary.docx]

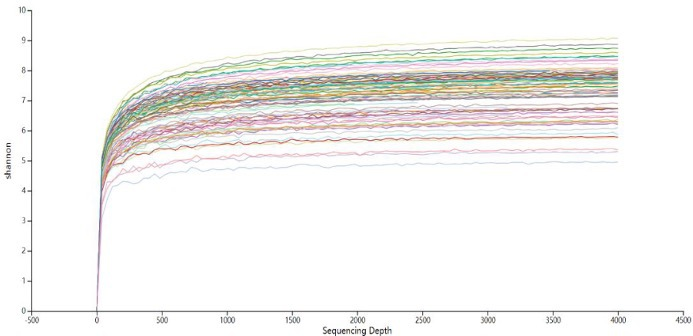


Figure S1 The dilution curves of the samples.


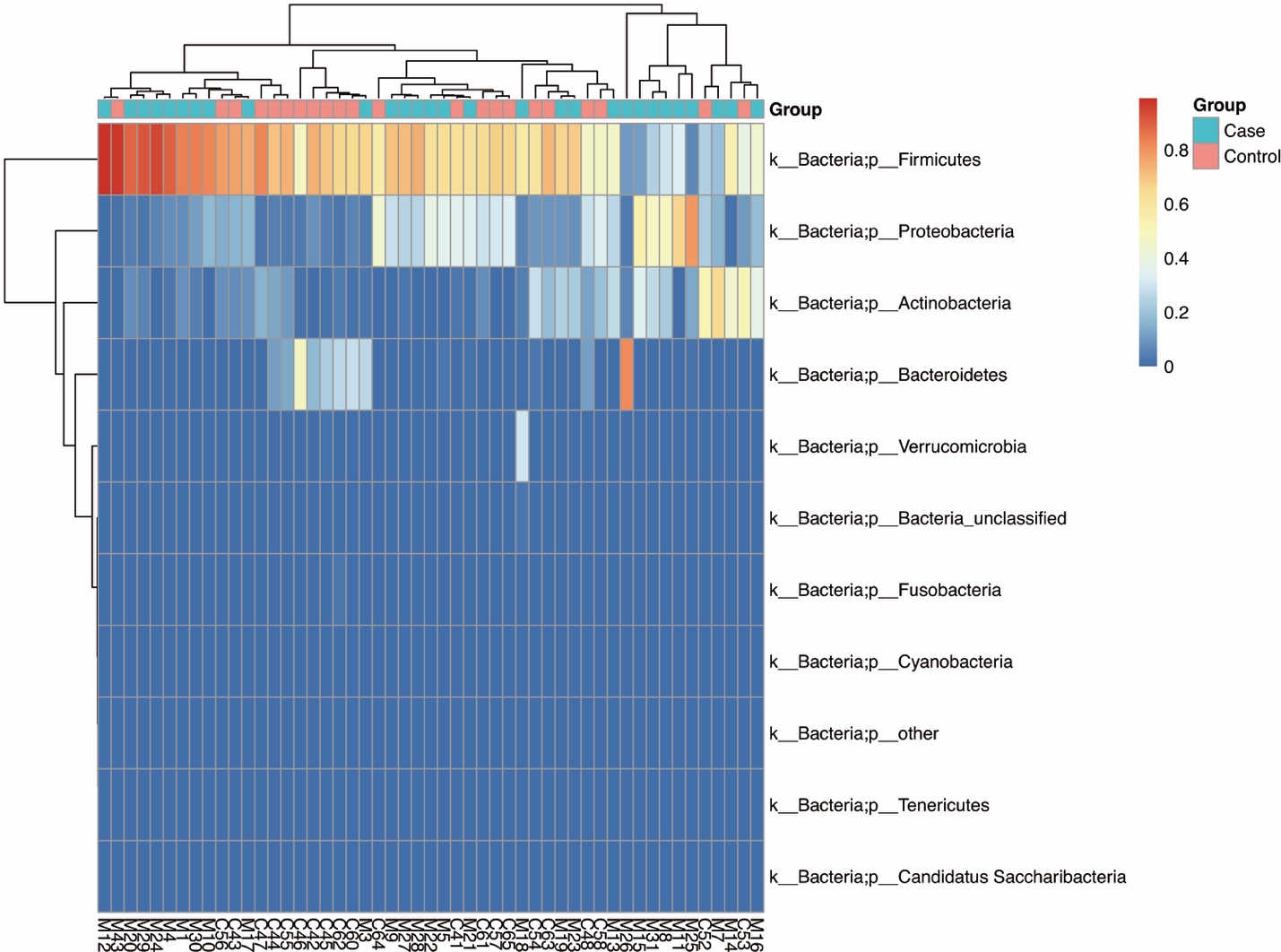


a


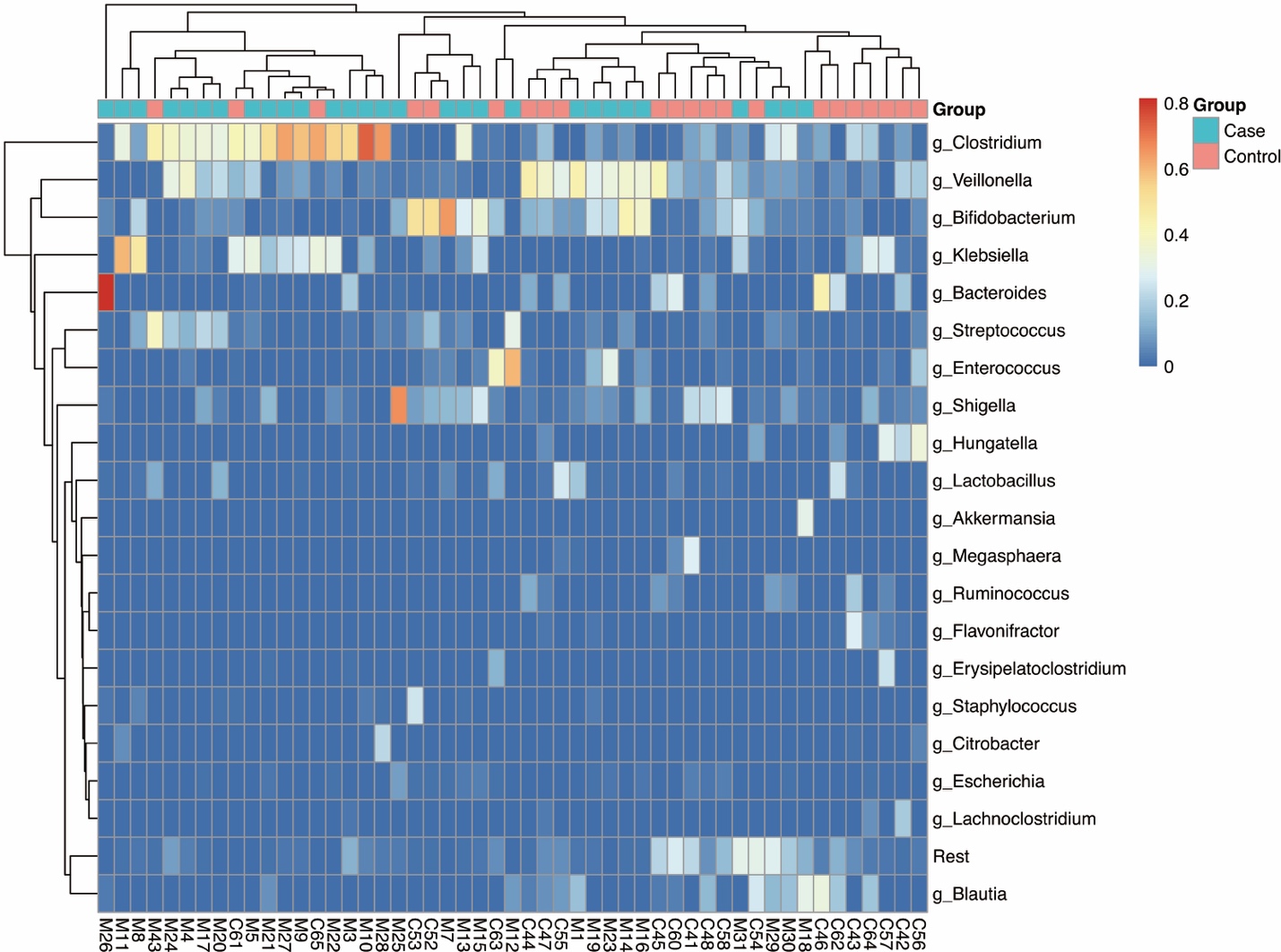


b


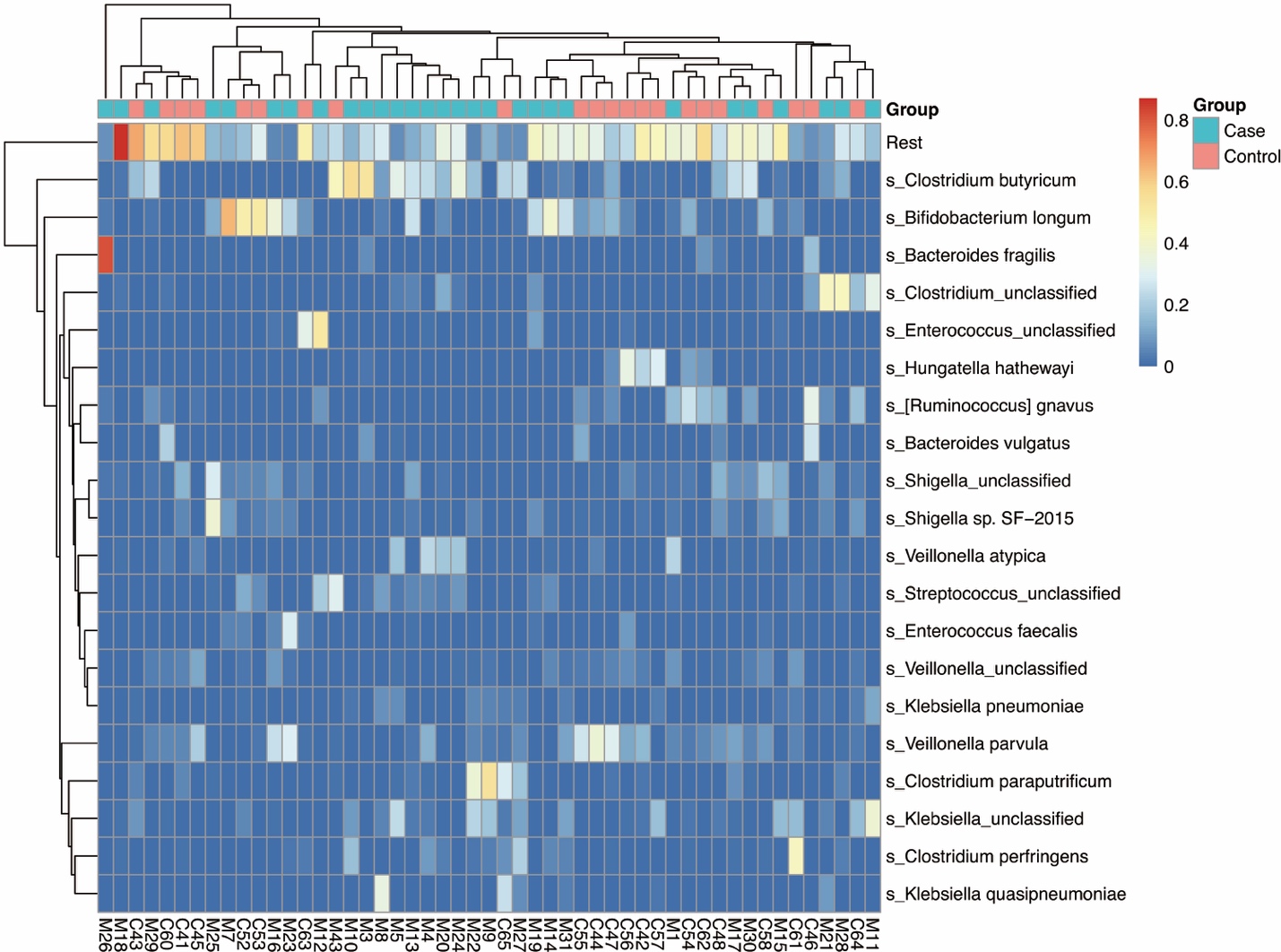


c

Figure S2-a,b,c The OTU heatmap of the relative abundance in two groups. a: phylum, b: genus, c: speices.


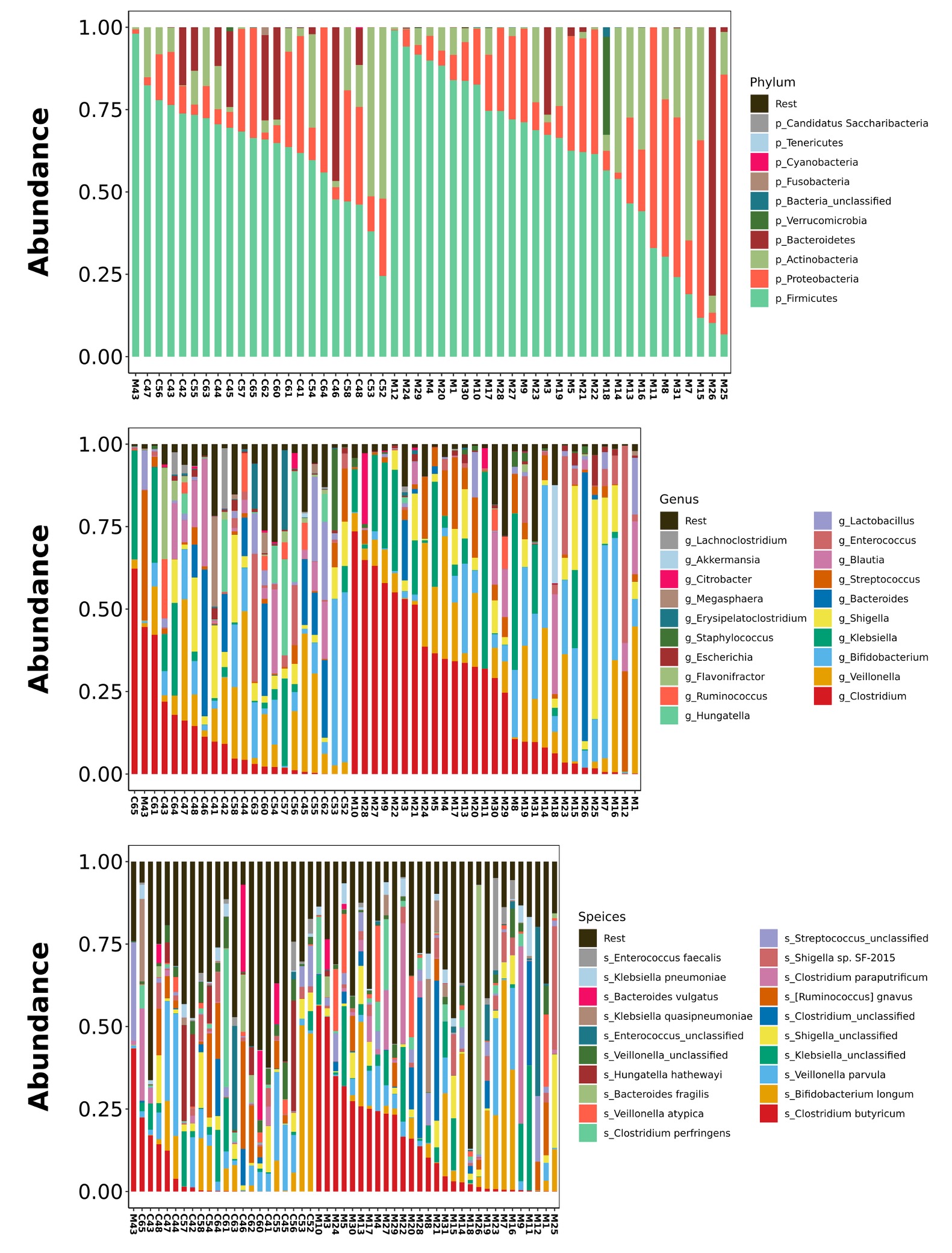


Figure S2-d Relative abundance distribution of two groups.


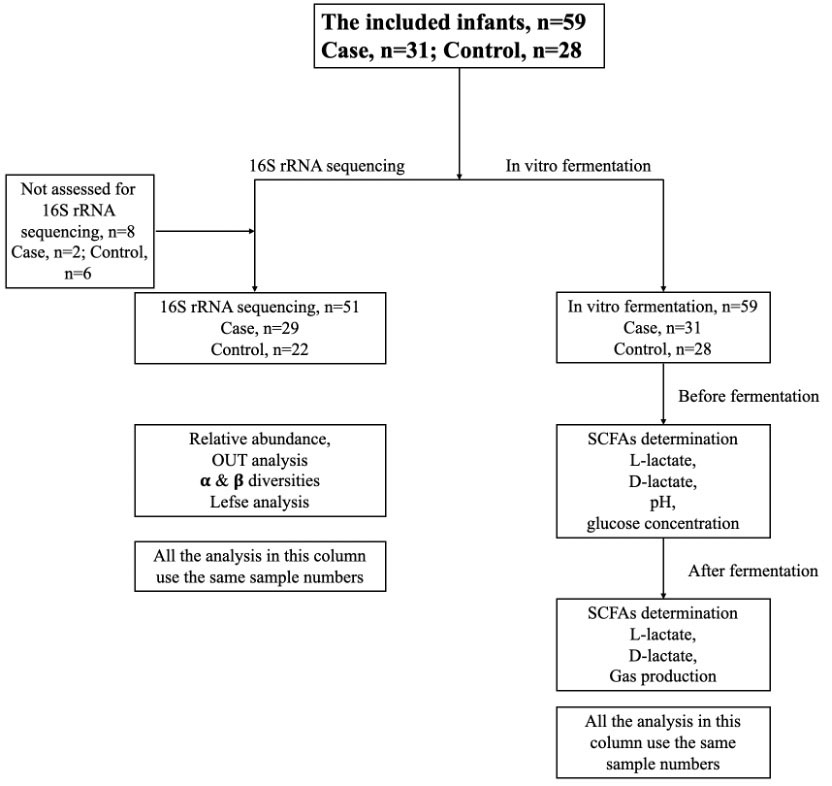


Figure S3
