# Supplementary figures and images for: Diagnostic and metabolic insights into secondary lactose intolerance in infants via fecal lactose quantification and gut microbiome profiling
Source: Front Immunol. 2026 Apr 21;17:1711945. doi: 10.3389/fimmu.2026.1711945 (PMC13139010; doi:10.3389/fimmu.2026.1711945)

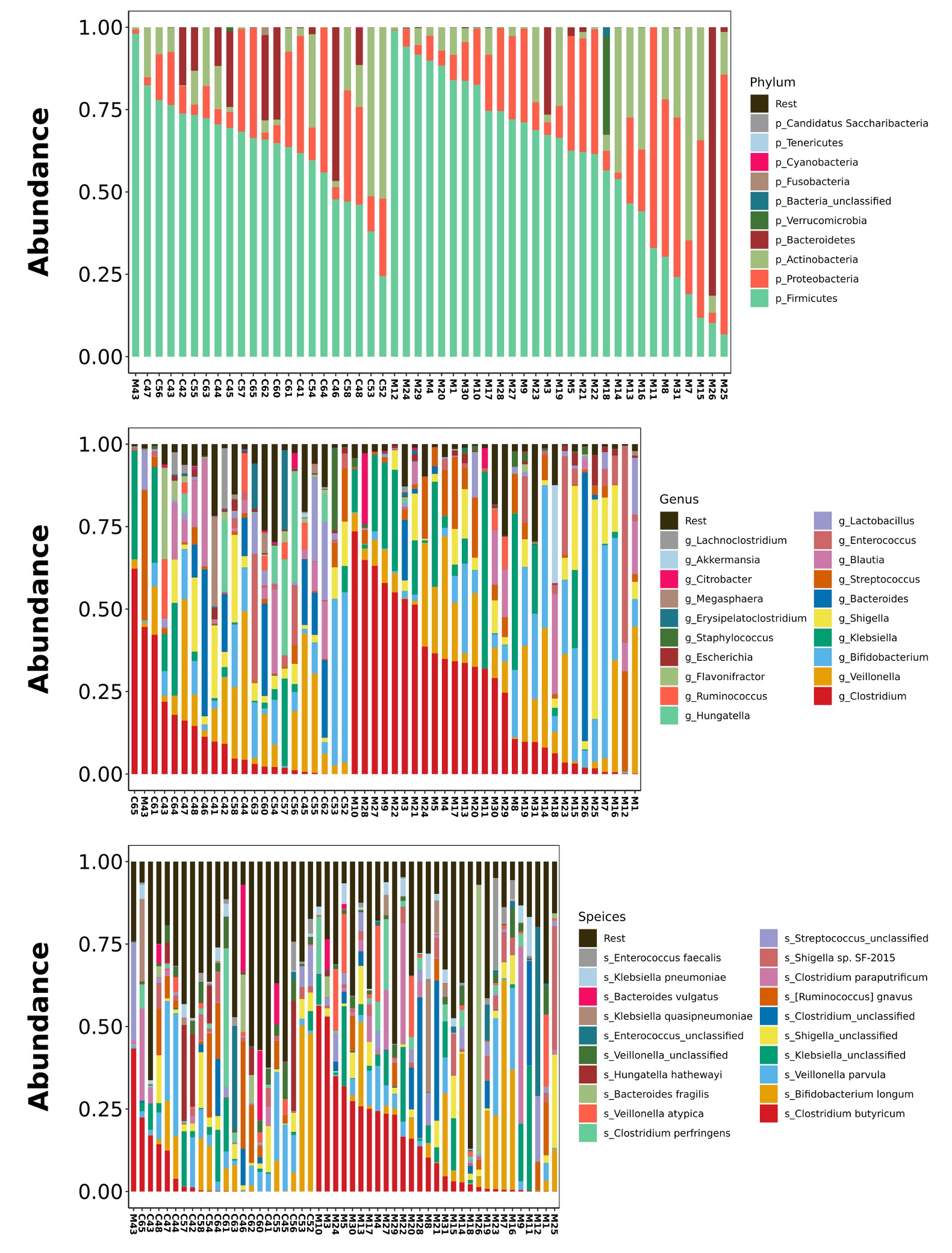

Supplement: Supplementary Figure 1 — The dilution curves of the samples. [file DataSheet1.zip › Data Sheet 1/Figure S2-d.jpg]

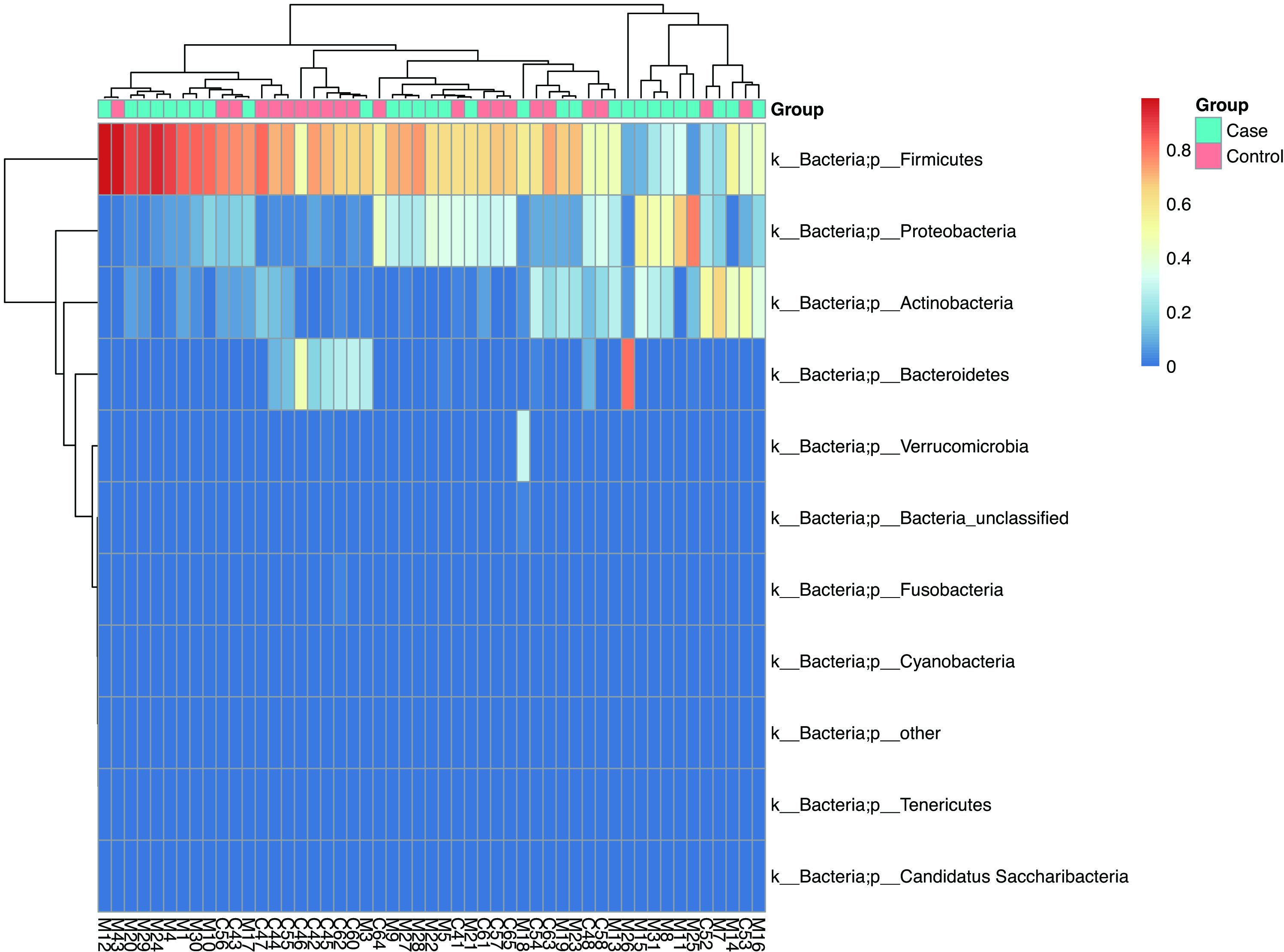

Supplement: Supplementary Figure 1 — The dilution curves of the samples. [file DataSheet1.zip › Data Sheet 1/Figure S2-a.jpg]

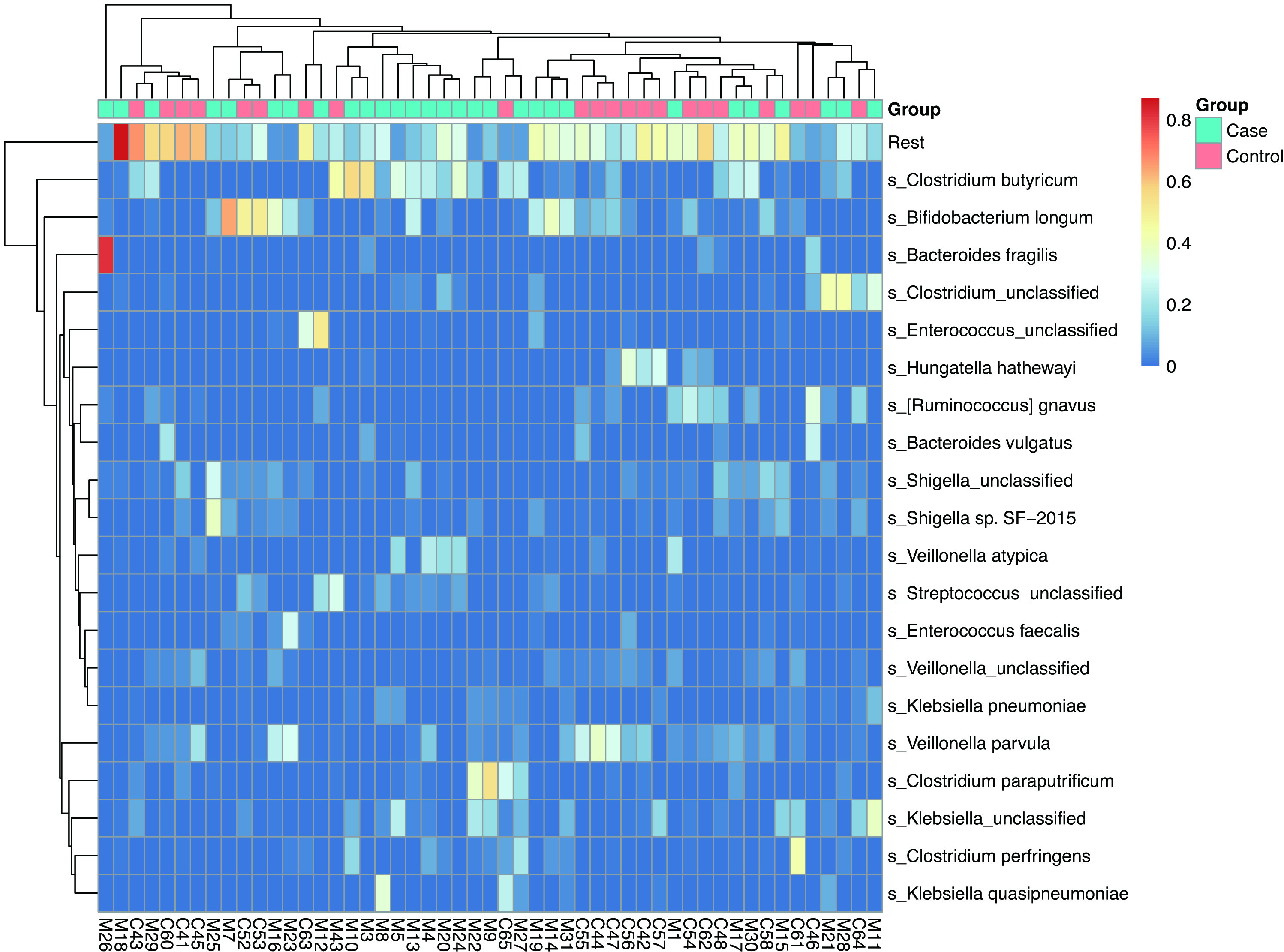

Supplement: Supplementary Figure 1 — The dilution curves of the samples. [file DataSheet1.zip › Data Sheet 1/Figure S2-c.jpg]

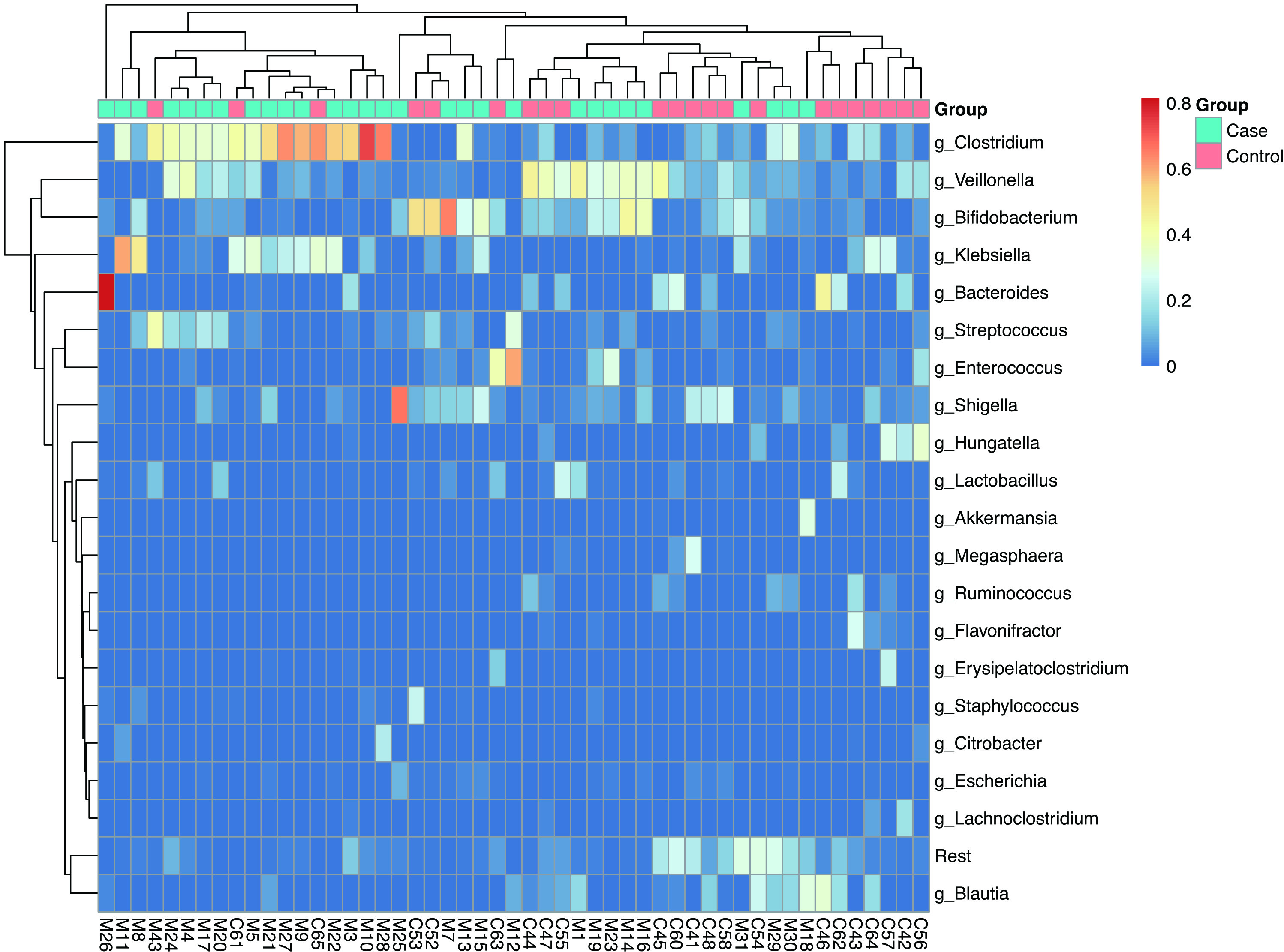

Supplement: Supplementary Figure 1 — The dilution curves of the samples. [file DataSheet1.zip › Data Sheet 1/Figure S2-b.jpg]

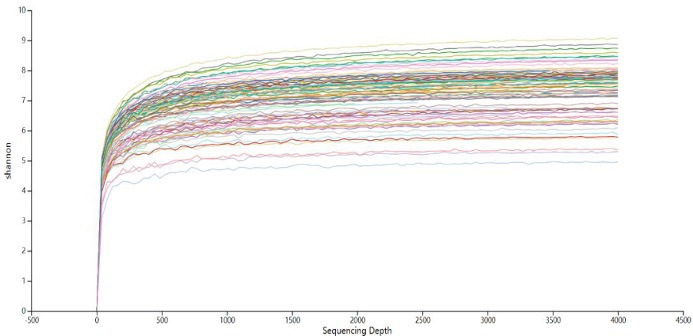

Supplement: Supplementary Figure 1 — The dilution curves of the samples. [file DataSheet1.zip › Data Sheet 1/Figure S1.jpg]
